# Supplementary material for: Digital Cell Sorter (DCS): a cell type identification, anomaly detection, and Hopfield landscapes toolkit for single-cell transcriptomics
Source: PeerJ. 2021 Jan 13;9:e10670. doi: 10.7717/peerj.10670 (PMC7811293; doi:10.7717/peerj.10670)
Supplement: Supplemental Information 1 [file peerj-09-10670-s001.pdf]

SUPPLEMENTARY MATERIALS

Q transformation on Hopfield landscapes

While Fig. 1 represents a landscape obtained without the matrix $Q_{\mu\nu}$, we show in Fig. S2 the Hopfield landscape obtained using the the matrix $Q_{\mu\nu}$ and the J_{ij} exactly as in Eq. 2. Note that, in this case, the Principal Component Analysis (PCA) components for the visualization has to be calculated on the transformed matrix $Q^{-1}\xi^T$, where ξ is the matrix of genes and attractors, instead of ξ^T as used in calculation of Fig. 1. From Fig. S2 it is clear how the use of the matrix Q_{ij} improves the separation between the attractor states, as compared to Fig. 1. The landscape is only using the first two principal components. Fig. S3 provides a complete representation of the overlaps between the cell attractors on the top non-trivial (non-zero) principal components obtained after the Q transformation.

Integration with existing algorithms

Batch correction

Data often contain noise associated to batch effects due to systematic experimental errors, collection procedures, and data handling (Tran et al. (2020); Lähnemann et al. (2020)). It is important to remove batch variations and technical noise in the data, yet preserving biologically relevant information. DCS includes a batch effect correction method, COMBAT, that has been developed specifically for microarray data, (Johnson et al. (2007); Stein et al. (2015)) but is also suitable for single cell transcriptomics data.

Handling of missing values is particularly important when dealing with batch effects correction in scRNA-seq. In our implementation of COMBAT we record the locations of missing values, i.e. zeros in the dataset, and perform the COMBAT transformation. Then, we replace with zeros any values that were missing and became non-zero after the transformation. We also replace with zeros any values that became negative.

Fig. S5 demonstrates how scRNA-seq data of plasma cells from the bone marrow aspirates of 12 patients (MM01-MM12) split into clusters corresponding to different batches. Processing the data with COMBAT and applying our procedure for missing values properly aligns the multi-dimensional data.

Clustering

DCS contains functions that implement different clustering methods:

1. Hierarchical clustering. This is in general a good choice for clustering single cell datasets (Luecken and Theis (2019)). However, this method becomes unfeasible when the size of the datasets goes beyond several tens of thousands cells. In this case DCS can use approximate methods such as k-means.

2. Network-based clustering methods. First a network of cells is constructed. Typically, it is a knn-graph (k nearest neighbors graph) with a cutoff k on the number of the nearest neighbors of each node. Then clusters are found using network-based algorithms such as modularity-based community detection (Newman (2010)).

3. Spectral co-clustering (Dhillon (2001)). Since this method is computationally demanding, it is recommended only for small subsets of the data. We have found that spectral co-clustering can accurately fine-separate cell sub-types when used with our cell type identification algorithms. In this case, we perform first a coarse clustering using one of the two methods above (e.g. to identify all T cells in the dataset) and then we use co-clustering to obtain cell subtypes (e.g. T CD8+, T CD4+, T memory, T naive, etc.).

Projection of high-dimensional data on 2D layout

Visualization of cell clusters can be done in DCS using different state-of-the-art methods to represent the results in a 2D layout:

1. t-SNE, Fig. S6 (a), a well-established nonlinear method that preserves local data structure (Maaten and Hinton (2008)).

2. PCA, based on the first two principal components (2nd PC vs. 1st PC) of the data, see Fig. S6 (b). This is a simple linear method that preserves the global structure of the data.

3. UMAP. This approach (McInnes et al. (2018)) has recently gained popularity because it preserves inter-cell distance in the dimensionality reduction procedure. This algorithm maintains the global structure and the continuity of the expression data. UMAP has been found to resolve cell populations and to produce equally meaningful representations compared with t-SNE (Becht et al. (2019)). Layouts produced with UMAP are more reproducible than other methods, notably more so than those from t-SNE. An example of visualization using UMAP on PBMC dataset is shown in Fig. S6 (c).

4. PHATE, showed in Fig. S6 (d). This recently developed method captures local and global structure using an information-geometric distance between data points (Moon et al. (2019)). PHATE has been found to reveal biological insights into cell developmental branches, including identification of previously undescribed subpopulations (Moon et al. (2019)).

Input gene expression data

The input gene expression data is expected in one of the following formats:

- Spreadsheet of comma-separated values (csv) containing a condensed matrix in a form ('cell', 'gene', 'expr'). If there are batches in the data, the matrix has to be of the form ('batch', 'cell', 'gene', 'expr'). Column order can be arbitrary.

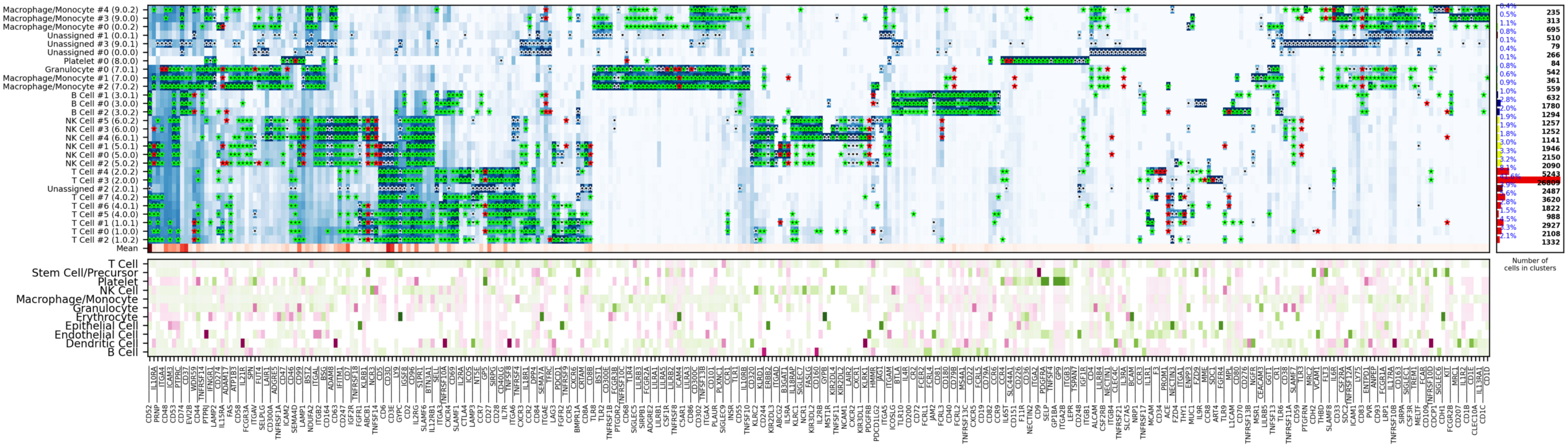

Figure S1. Top panel: heatmap of the average gene expression for each cluster, with darker blue corresponding to higher expression values. Green stars indicate supporting markers of the assigned cell type, red stars are contradicting, and white stars are neither supporting nor contradicting but significantly expressed. The assigned cell type is indicated on the left and combined with the cluster number id. Bottom panel: normalized marker cell type matrix $\tilde{M}$ with dark green (rose) indicating unique positive (negative) markers. Right panel:total numbers of cell for each type and cluster.

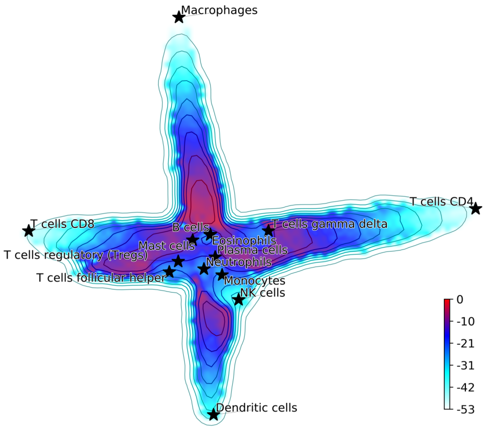

Figure S2. Hopfield attractor landscape visualization. The points are colored according to their Hopfield energy. The Q transformation has improved the separation between the attractor states compared to Fig. 1.

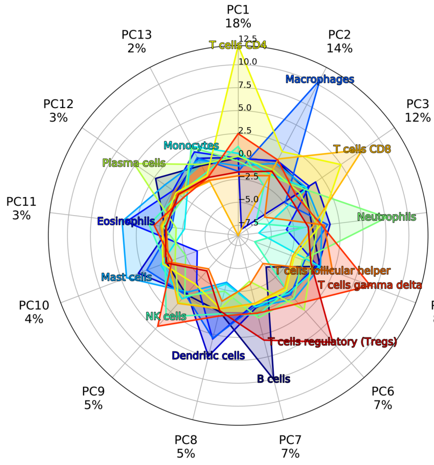

Figure S3. Plot of the projection of different cell attractors on PCA components obtained after the Q transformation.

- Spreadsheet of comma-separated values csv where rows are genes, columns are cells with gene expression counts. If there are batches in the data the spreadsheet the first row should be 'batch' and the second 'cell'.
- Pandas DataFrame where axis 0 is genes and axis 1 are cells. If there are batched in the data, then the index of axis 1 should have two levels, e.g. ('batch', 'cell'), with the first level indicating patient, batch or experiment where that cell was sequenced, and the second level containing cell barcodes.
- Pandas Series where the index should have two levels, e.g. ('cell', 'gene'). If there are batched in the data the first level should be indicating patient, batch or experiment where that cell was sequenced, the second level cell barcodes, and the third level gene names.

During and after processing data storage is implemented using Hierarchical Data Format (HDF).

Miscellaneous analysis tools

The optimized performance of our modular DCS software allows for an efficient processing of large single cell datasets. The documentation of our software is built with Sphinx at ReadTheDocs.org. Any changes to source code and python code docstrings are automatically reflected at ReadTheDocs.org, and a new version of the documentation is built.

In DCS we have implemented numerous querying functions for an efficient extraction of cells based on a specific cluster or cell type. This functionality allows for an easy selection of data subsets for further analysis.

A specialized function in DCS provides across clusters comparison via a two-tailed t-test plot of individual genes. See the example of CD4 expression in PBMC data in the Fig. S7.

Finally, DCS includes a function representing in a pie chart the role of different markers in a direct comparison between two cell types. Fig. S8 shows an example of output for T cells versus NK cells markers. This function can be useful, for instance, to make the final decision on a cluster for which the cell type assignment has provided two possible cell types.

REFERENCES

Becht, E., McInnes, L., Healy, J., Dutertre, C.-A., Kwok, I. W. H., Ng, L. G., Ginhoux, F., and Newell, E. W. (2019). Dimensionality reduction for visualizing single-cell data using UMAP. *Nature Biotechnology*, 37(1):38–44. Number: 1 Publisher: Nature Publishing Group.

Dhillon, I. S. (2001). Co-clustering documents and words using bipartite spectral graph partitioning. In *Proceedings of the seventh ACM SIGKDD international conference on Knowledge discovery and data mining*, KDD '01, pages 269–274, San Francisco, California. Association for Computing Machinery.

Johnson, W. E., Li, C., and Rabinovic, A. (2007). Adjusting batch effects in microarray expression data using empirical Bayes methods. *Biostatistics*, 8(1):118–127. Publisher: Oxford Academic.

Lähnemann, D., Köster, J., Szczurek, E., McCarthy, D. J., Hicks, S. C., Robinson, M. D., Vallejos, C. A., Campbell, K. R., Beerenwinkel, N., Mahfouz, A., Pinello, L., Skums, P., Stamatakis, A., Attolini, C. S.-O., Aparicio, S., Baaijens, J., Balvert, M., Barbanson, B. d., Cappuccio, A., Corleone, G., Dutilh, B. E., Florescu, M., Guryev, V., Holmer, R., Jahn, K., Lobo, T. J., Keizer, E. M., Khatri, I., Kielbasa, S. M., Korbel, J. O., Kozlov, A. M., Kuo, T.-H., Lelieveldt, B. P., Mandoiu, I. I., Marioni, J. C., Marschall, T., Mölder, F., Niknejad, A., Raczkowski, L., Reinders, M., Ridder, J. d., Saliba, A.-E., Somarakis, A., Stegle, O., Theis, F. J., Yang, H., Zelikovsky, A., McHardy, A. C., Raphael, B. J., Shah, S. P., and Schönhuth, A. (2020). Eleven grand

Table S1. Metadata of samples used in validation of anomaly detection.

Accession	GSM3169075 SRA713577 SRS3363004	GSM3402081 SRA784242 SRS3822686	GSM3396161 SRA779509 SRS3805245	GSM3330560 SRA749327 SRS3693909	GSM3589360 SRA843432 SRS4322341
Sequencing protocol	10x chromium	10x chromium	10x chromium	10x chromium	10x chromium
Sequencing instrument	Illumina HiSeq 2500	Illumina HiSeq 2500	Illumina HiSeq 3000	Illumina HiSeq 2500	NextSeq 500
Tissue origin of the sample	PBMC	Testicular cells	Bone marrow	Merkel cell carcinoma	Kaposi's sarcoma
Number of sequenced genes	27483	28403	30594	28924	26224
Number of sequenced cells	6008	6361	8307	6696	5140
Number of QC-passed cells	3167	1501	5928	5347	3318
Median of genes per cell	1178	1100	1106	1272	1184
Used cell clusters	#0: 666 T cells, #1: 564 T cells, #2: 407 Monocytes	#6: 70 Endothelial cells	#0: 928 T cells	#8: 78 cells	#8: 43 cells

challenges in single-cell data science. *Genome Biology*, 21(1):31.

Luecken, M. D. and Theis, F. J. (2019). Current best practices in single-cell RNA-seq analysis: a tutorial. *Molecular Systems Biology*, 15(6):e8746.

Maaten, L. v. d. and Hinton, G. (2008). Visualizing data using t-SNE. *Journal of Machine Learning Research*, 9(Nov):2579–2605.

McInnes, L., Healy, J., and Melville, J. (2018). UMAP: Uniform Manifold Approximation and Projection for Dimension Reduction. *arXiv:1802.03426 [cs, stat]*. arXiv: 1802.03426.

Moon, K. R., van Dijk, D., Wang, Z., Gigante, S., Burkhardt, D. B., Chen, W. S., Yim, K., Elzen, A. v. d., Hirn, M. J., Coifman, R. R., Ivanova, N. B., Wolf, G., and Krishnaswamy, S. (2019). Visualizing structure and transitions in high-dimensional biological data. *Nature Biotechnology*, 37(12):1482–1492. Number: 12 Publisher: Nature Publishing Group.

Newman, M. (2010). *Networks: An Introduction*. Oxford University Press.

Stein, C. K., Qu, P., Epstein, J., Buros, A., Rosenthal, A., Crowley, J., Morgan, G., and Barlogie, B. (2015). Removing batch effects from purified plasma cell gene expression microarrays with modified ComBat. *BMC Bioinformatics*, 16(1):63.

Tran, H. T. N., Ang, K. S., Chevrier, M., Zhang, X., Lee, N. Y. S., Goh, M., and Chen, J. (2020). A benchmark of batch-effect correction methods for single-cell RNA sequencing data. *Genome Biology*, 21(1):12.

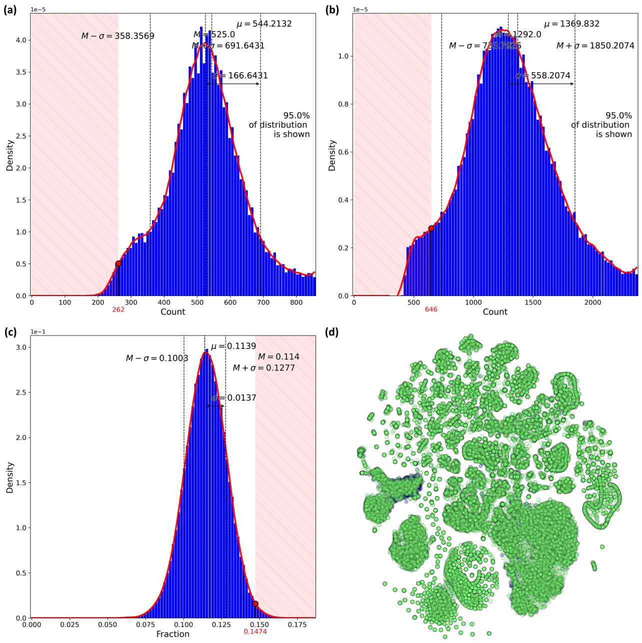

Figure S4. Quality Control. (a) Histogram of the number of unique genes in each cell. The red line is the spline fit of the distribution. The cutoff is determined as 50% of the median of the distribution. Cells that have gene counts in the red shaded area, i.e. below the cutoff, are discarded before clustering; (b) Same as in (a) for the number of total reads per cell; (c) Histogram of fraction of mitochondrial genes in each cell. Values in the red shaded area are discarded; (d) t-SNE layout of the analyzed data where the dark blue points are cells discarded as not passing quality criteria in (a)-(c).

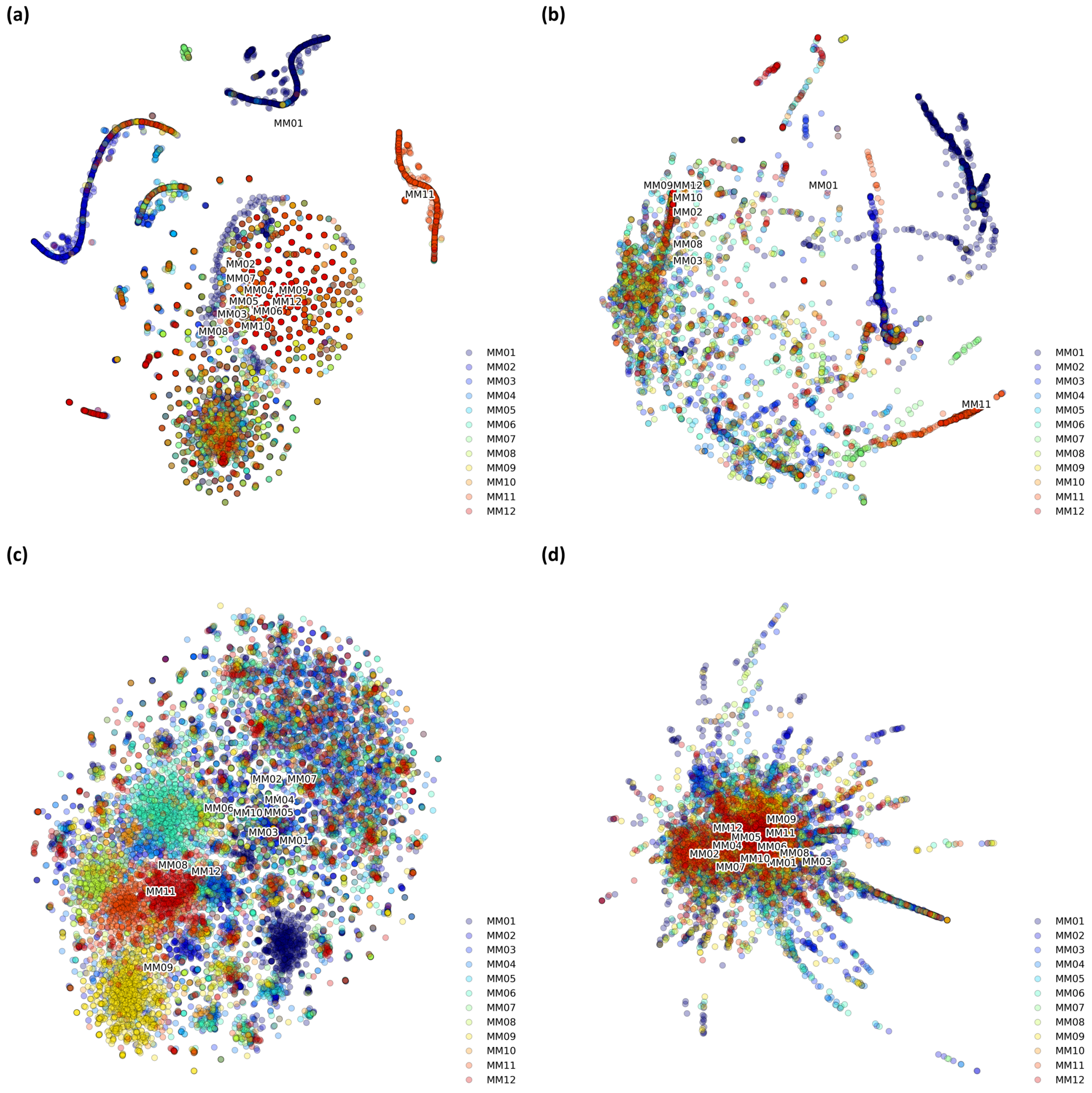

Figure S5. scRNA-seq data of Bone Marrow plasma cells from 12 patients (MM01-MM12) split into clusters clearly showing batch-effects in (a) t-SNE layout and (b) PHATE layout. Processing these data sets with COMBAT aligns the multi-dimensional transcriptomics data mitigating batch effects as seen in (c) t-SNE layout and (d) PHATE layout.

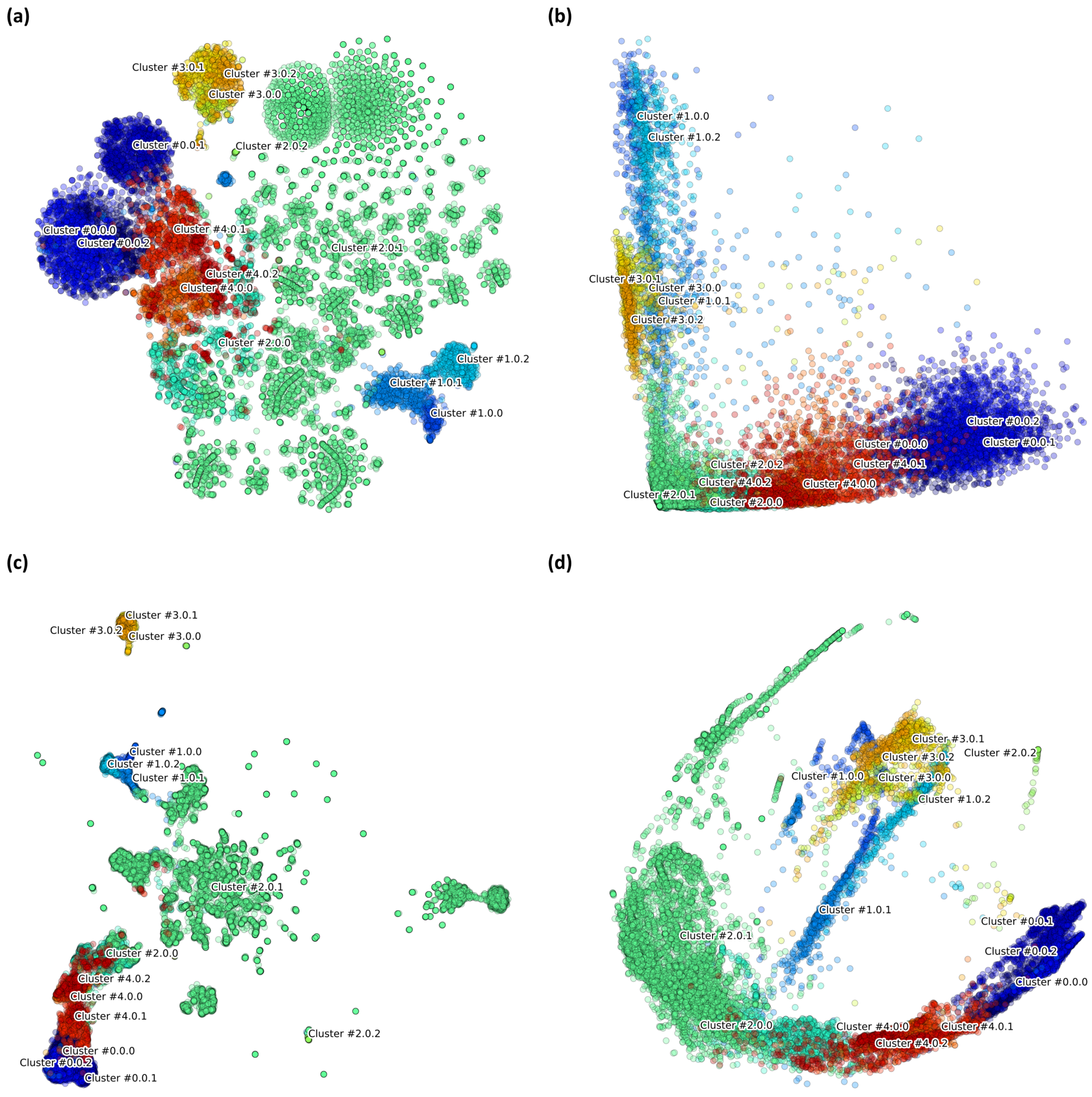

Figure S6. Two-dimensional projection of PBMC scRNA-seq data on (a) t-SNE, (b) two largest principal components of PCA, (c) PHATE layout, and (d) UMAP layout.

Table S2. Inter- and intra-cluster distances for clusters L, M and O, and the Silhouette score for each of the clusters and all the cells. The measures are the average of a hundred independent realizations with a new set of cell in cluster O every time.

	Measure	Cluster	Myeloid (M)	Lymphoid (L)	Other (O)	All ($M+L+O$)
**Normal endothelial cells**	*Average distance*	Myeloid (M)	15.99	22.96	41.66	
		Lymphoid (L)		8.01	36.24	
		Other (O)			31.81	
		All ($M+L+O$)				14.65
	Silhouette score		0.30	0.65	0.13	0.56
**Normal bone marrow lymphocytes**	Measure	Cluster	Myeloid (M)	Lymphoid (L)	Other (O)	All ($M+L+O$)
	Average distance	Myeloid (M)	16.63	23.59	26.10	
		Lymphoid (L)		8.80	15.59	
		Other (O)			13.70	
		All ($M+L+O$)				14.88
	Silhouette score		0.29	0.44	0.12	0.40
**Kaposi's sarcoma cells**	Measure	Cluster	Myeloid (M)	Lymphoid (L)	Other (O)	All ($M+L+O$)
	Average distance	Myeloid (M)	16.55	23.57	30.49	
		Lymphoid (L)		8.59	20.31	
		Other (O)			16.82	
		All ($M+L+O$)				14.86
	Silhouette score		0.30	0.58	0.18	0.50
**Merkel cell carcinoma cells**	Measure	Cluster	Myeloid (M)	Lymphoid (L)	Other (O)	All ($M+L+O$)
	Average distance	Myeloid (M)	14.80	22.11	39.70	
		Lymphoid (L)		7.34	33.18	
		Other (O)			35.21	
		All ($M+L+O$)				13.82
	Silhouette score		0.33	0.67	-0.05	0.58

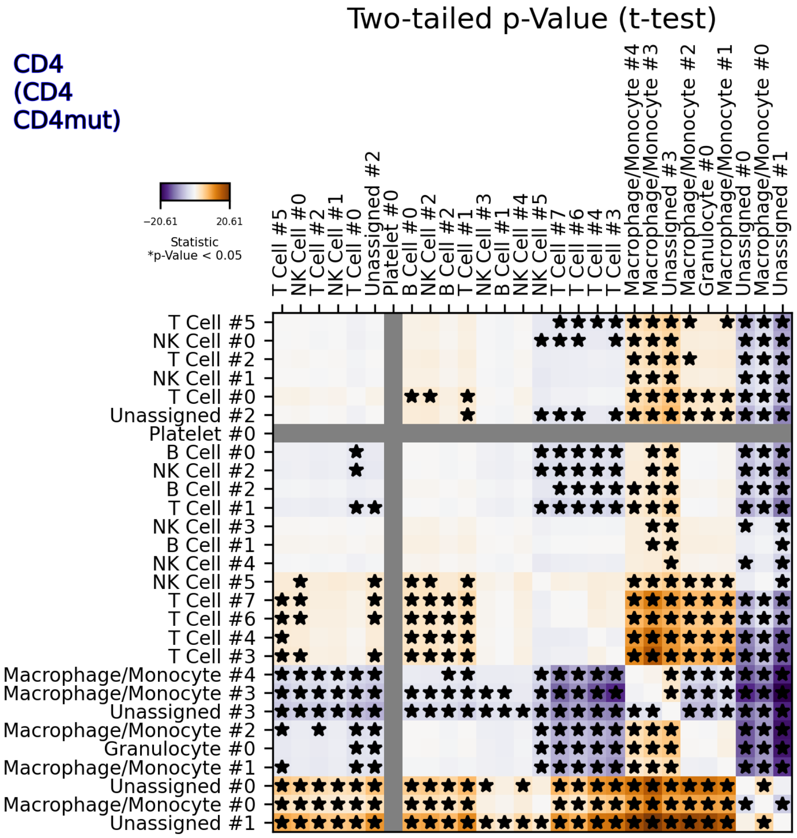

Figure S7. Example of two-tailed t-test analysis for the CD4 gene expression in the 68k PBMC dataset. Black stars denote where this gene is significantly expressed in a two-cluster cross-comparison.

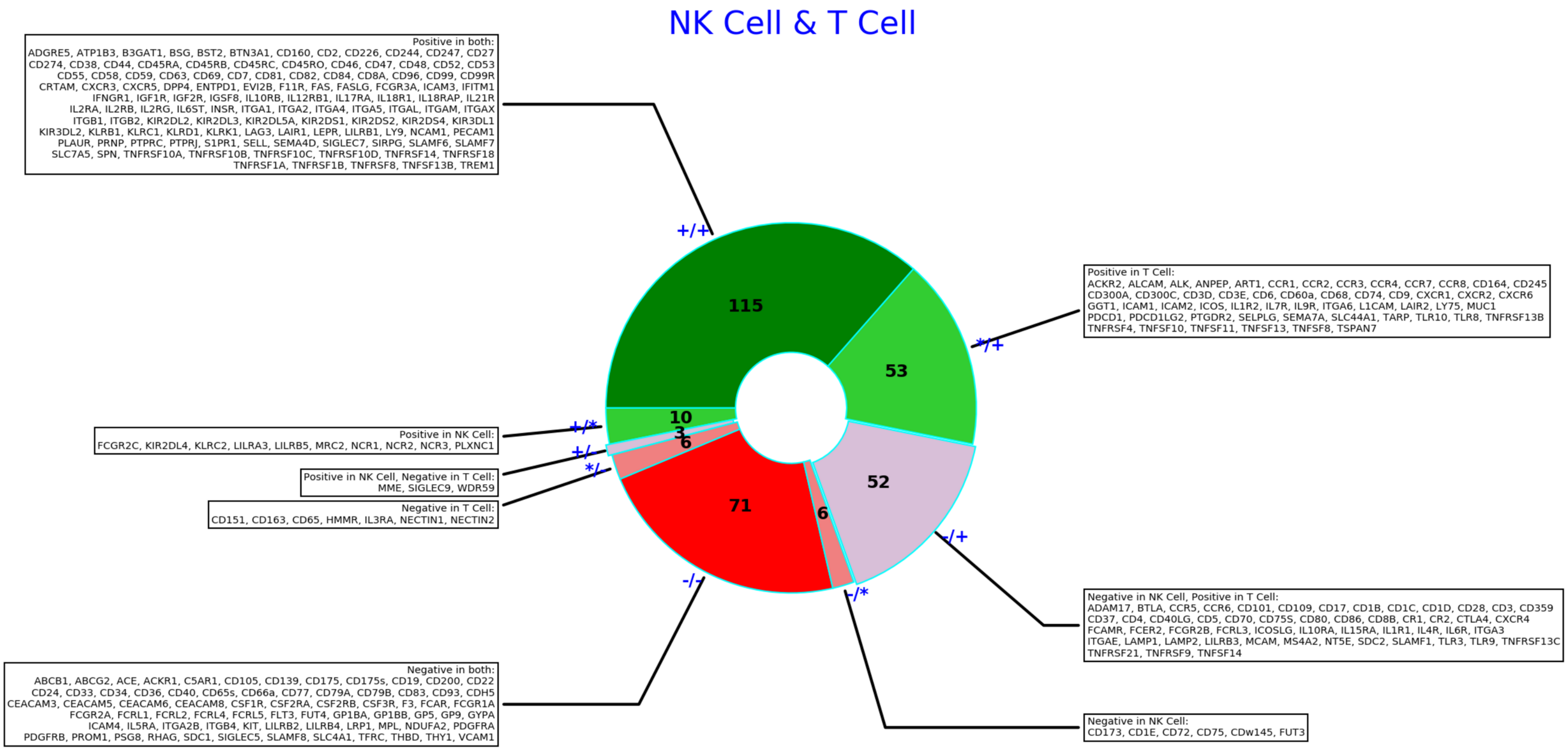

Figure S8. Summary of T cells and NK cells markers from the "CD Marker Handbook". The dark green sector shows markers expected to be found in both cell types, the light green sector contains markers expressed in one of the cell types. The dark red sector is for markers that are not expected to be significantly expressed in either cell types, and light red is for markers that should not be significantly expressed in one of the two cell types. Grey-shaded sector contain markers that are expected to be expressed in one of the cell type and non-expressed in the other. The number in the center is the total number of known markers for these two cell types.
