# Supplementary material for: Digital Cell Sorter (DCS): a cell type identification, anomaly detection, and Hopfield landscapes toolkit for single-cell transcriptomics
Source: PeerJ. 2021 Jan 13;9:e10670. doi: 10.7717/peerj.10670 (PMC7811293; doi:10.7717/peerj.10670)
Supplement: Supplemental Information 2 [file peerj-09-10670-s002.pdf]

DigitalCellSorter Documentation

Release 1.3.7

S. Domanskyi, A. Szedlak, N. T Hawkins, J. Wang, T. Bertus, A. Ha

Nov 26, 2020

CONTENTS:

1 Overview **3**

1.1 Publications . . . 3

1.2 Description of the package functionality . . . 3

1.3 Versions change log . . . 3

2 Getting Started **7**

2.1 Installation . . . 7

2.2 Loading the package . . . 7

3 User Functions **9**

3.1 Primary tools . . . 10

3.2 Extraction tools . . . 13

3.3 Visualization tools . . . 16

4 Core class **29**

5 Visualization functions API **47**

5.1 Cell type markers pie plot . . . 47

5.2 Projection plot . . . 48

5.3 Marker subplots . . . 48

5.4 Quality control histogram plot . . . 49

5.5 Histogram null distribution plot . . . 50

5.6 Sankey diagram . . . 52

5.7 Stacked bar plot . . . 53

5.8 Annotation Results Matrix plot . . . 53

5.9 Marker expression plot . . . 55

5.10 t-test plot . . . 55

5.11 Plot of new markers . . . 56

6 Generic functions **57**

7 Dependencies **59**

8 Data preparation **61**

8.1 Output from kallisto-bustools (kp-python) . . . 61

8.2 Output from CellRanger . . . 62

8.3 Import from kallisto-bustools (kp-python) . . . 63

8.4 Import from CellRanger . . . 63

8.5 Function readMTXdata . . . 63

8.6 Human Cell Atlas tools . . . 64

9 Input Data Format 67

10 Demo 69

11 Indices and tables 71

Python Module Index 73

Index 75

Identification of hematological cell types from heterogeneous single cell RNA-seq data.

Polled Digital Cell Sorter (p-DCS): Automatic identification of hematological cell types from single cell RNA-sequencing clusters. Sergii Domanskyi, Anthony Szedlak, Nathaniel T Hawkins, Jiayin Wang, Giovanni Paternostro & Carlo Piermarocchi, *BMC Bioinformatics* volume 20, Article number: 369 (**2019**) https://doi.org/10.1186/s12859-019-2951-x

CHAPTER

ONE

OVERVIEW

1.1 Publications

Identification of hematological cell types from heterogeneous single cell RNA-seq data.

Polled Digital Cell Sorter (p-DCS): Automatic identification of hematological cell types from single cell RNA-sequencing clusters. Sergii Domanskyi, Anthony Szedlak, Nathaniel T Hawkins, Jiayin Wang, Giovanni Paternostro & Carlo Piermarocchi, *BMC Bioinformatics* volume 20, Article number: 369 (**2019**) https://doi.org/10.1186/s12859-019-2951-x

1.2 Description of the package functionality

The main class of DigitalCellSorter. The class includes tools for:

1. **Pre-preprocessing** of single cell RNA sequencing data
2. **Quality control**
3. **Batch effects correction**
4. **Cells anomaly score evaluation**
5. **Dimensionality reduction**
6. **Clustering**
7. **Annotation of cell types**
8. **Vizualization**
9. **Post-processing**

1.3 Versions change log

- **1.3.7**
  - Added a function to import data from kallisto-bustools and cellranger
  - Updated documentation
- **1.3.6**
  - Added quick-demo materials
- **1.3.5**

  - Miscellaneous code improvements and bug fixes
- **1.3.4.0-1.3.4.11**
  - Integrated plotly offline figure saving (when orca is unavailable)
  - Added Quality Control pre-cut
- **1.3.2**
  - Added Hopfield landscape visuzlization capability
  - Added network of underlying biological gene-gene interaction to the Hopfield annotaiton scheme
- **1.3.1**
  - Minor API modifications
- **1.3.0**
  - Modified pDCS algorithm for cell type identification to account for markers that should not be expressed in a given cell type (negative markers)
  - Modified pDCS celltype/marker matrix normalization
  - Modified pDCS algorithm account for low quality scores
  - Added Hopfield classifier for cell type annotation
  - Added ratio method for cell type annotation
  - Added options for consensus cell type annotation
  - Added cell markers pie summary function and plot
  - Added t-test for individual gene plot
  - Added several new user functions, for efficient and flexible extraction of cells, genes, clusters, etc.
  - Added anomaly score calculation and visualization
  - Refactored function for extraction of new markers based on cell type annotations to separate it from function process() of class DigitalCellSorter
  - Optimized implementation (for higher performance) of various function of this package
  - Detailed visualization functions API
  - Incorporated different clustering methods in addition to the widely-utilized hierarchical clustering
  - Incorporated several types of high-dimensional data projection methods, such as efficient t-SNE, UMAP and simple PCA components.
  - Extended options for input data format
  - Included a set of functions to load data from Human Cell Atlas (HCA) and prepare it for processing
- **1.2.3**
  - API updates, documentation updates
- **1.2.1**
  - Minor updates, reshaped DigitalCellSorter into a stand-alone package
- **1.2.0**
  - More features, better runtime efficiency
- **1.1**

    - Updated method, signature matrices

- **1.0**
    - First Release

CHAPTER

TWO

GETTING STARTED

These instructions will get you a copy of the project up and running on your machine for data analysis, development or testing purposes.

2.1 Installation

Install of the latest release of `DigitalCellSorter`:

```
$ pip install DigitalCellSorter
```

For detailed instructions and other ways to install `DigitalCellSorter` as wells as list of optional packages and instructions on how to install them see **Prerequisites** section at https://github.com/sdomanskyi/DigitalCellSorter

2.2 Loading the package

In your script import the package:

```
import DigitalCellSorter
```

Create an instance of `class DigitalCellSorter`. Here, for simplicity, we use Default parameter values:

```
DCS = DigitalCellSorter.DigitalCellSorter()
```

CHAPTER

THREE

USER FUNCTIONS

User functions from **DigitalCellSorter.core.DigitalCellSorter** class.

Note: All of the tools listed below in this section are intended to use from an instance of a `DigitalCellSorter` class. For example:

```
DCS = DigitalCellSorter.DigitalCellSorter()

DCS.dataName = 'my_data_name'
DCS.saveDir = os.path.join(os.path.dirname(__file__), 'output', DCS.dataName, '')

data = DCS.prepare(raw_data)

DCS.process(DCS.prepare(data))

DCS.makeIndividualGeneExpressionPlot('CCL5')

DCS.makeIndividualGeneTtestPlot('CCL5', analyzeBy='celltype')

cells = DCS.getCells(celltype='T cell')
DCS.makeAnomalyScoresPlot(cells)

# ...
```

Direct use of function from where they are stored may result in undefined behavior.

Description of the package functionality

The main class of DigitalCellSorter. The class includes tools for:

1. **Pre-preprocessing** of single cell RNA sequencing data
2. **Quality control**
3. **Batch effects correction**
4. **Cells anomaly score evaluation**
5. **Dimensionality reduction**
6. **Clustering**
7. **Annotation of cell types**
8. **Vizualization**
9. **Post-processing**

3.1 Primary tools

Primary tools are used for pre-processing of the input data, quality control, batch correction, dimensionality reduction, clustering and cell type annotation.

Note: We reccomend to use only functions `prepare()`, `process()`, and `visualize()` of the Primary tools. All processing workflow is contained within `process()`. If you wish to modify the workflow use the other components of the Primary tools, such as `cluster()`, `project()` etc.

References to DigitalCellSorter class:

`prepare`(obj)	Prepare pandas.DataFrame for input to function process() If input is pd.DataFrame validate the input whether it has correct structure.
`convert`([nameFrom, nameTo])	Convert index to hugo names, if any names in the index are duplicated, remove duplicates
`clean`()	Clean pandas.DataFrame: validate index, remove index duplicates, replace missing with zeros, remove all-zero rows and columns
`project`([PCAonly, do_fast_tsne])	Project pandas.DataFrame to lower dimensions
`cluster`()	Cluster PCA-reduced data into a desired number of clusters
`annotate`([mapNonexpressedCelltypes])	Produce cluster voting results, annotate cell types, and update marker expression with cell type labels
`process`([dataIsNormalized, cleanData])	Process data before using any annotation of visualization functions
`visualize`()	Aggregate of visualization tools of this class.

Function `prepare()`: prepare input data for function `process()`

`DigitalCellSorter.`**`prepare`**(*obj*)

Prepare pandas.DataFrame for input to function process() If input is pd.DataFrame validate the input whether it has correct structure.

Parameters:

obj: str, pandas.DataFrame, pandas.Series Expression data in a form of pandas.DataFrame, pandas.Series, or name and path to a csv file with data

Returns: None

Usage: DCS = DigitalCellSorter.DigitalCellSorter()

dDCS.preapre('data.csv')

Function `convert()`: convert gene index of a DataFrame prepared by function `prepare()` from one naming

convention to another

`DigitalCellSorter.`**`convert`**`(`*nameFrom=None*, *nameTo=None*, ***kwargs*`)`

Convert index to hugo names, if any names in the index are duplicated, remove duplicates

Parameters:

nameFrom: str, Default 'alias' Gene name type to convert from

nameTo: str, Default 'hugo' Gene name type to convert to

Any parameters that function 'mergeIndexDuplicates' can accept

Returns: None

Usage: DCS = DigitalCellSorter.DigitalCellSorter()

DCS.convertIndex()

Function `clean()`: validate index, replace missing with zeros, remove all-zero rows and columns of a DataFrame

`DigitalCellSorter.`**`clean`**`()`

Clean pandas.DataFrame: validate index, remove index duplicates, replace missing with zeros, remove all-zero rows and columns

Parameters: None

Returns: None

Usage: DCS = DigitalCellSorter.DigitalCellSorter()

DCS.clean()

Function `normalize()`: rescale all cells, log-transform data, remove constant genes, and sort index of a DataFrame

`DigitalCellSorter.`**`normalize`**`(`*median=None*`)`

Normalize pandas.DataFrame: rescale all cells, log-transform data, remove constant genes, sort index

Parameters:

median: float, Default None Scale factor, if not provided will be computed as median across all cells in data

Returns: None

Usage: DCS = DigitalCellSorter.DigitalCellSorter()

DCS.normalize()

Function `project()`: project data to lower dimensions

`DigitalCellSorter.`**`project`**`(`*PCAonly=False*, *do_fast_tsne=True*`)`

Project pandas.DataFrame to lower dimensions

Parameters:

PCAonly: boolean, Default False Perform Principal component analysis only

do_fast_tsne: boolean, Default True Do FI-tSNE instead of "exact" tSNE This option is ignored if layout is not 'TSNE'

Returns:

tuple Processed data

Usage: DCS = DigitalCellSorter.DigitalCellSorter()

xPCA, PCs, tSNE = DCS.project()

Function `cluster()`: cluster PCA-reduced data into a desired number of clusters

`DigitalCellSorter.`**`cluster`**`()`

Cluster PCA-reduced data into a desired number of clusters

Parameters: None

Returns: None

Usage: DCS = DigitalCellSorter.DigitalCellSorter()

DCS.cluster()

Function `annotate()`: produce cluster voting results

`DigitalCellSorter.`**`annotate`**`(`*mapNonexpressedCelltypes=True*`)`

Produce cluster voting results, annotate cell types, and update marker expression with cell type labels

Parameters:

mapNonexpressedCelltypes: boolean, Default True If True then cell types coloring will be consistent across all datasets, regardless what cell types are annotated in all datasets for a given input marker list file.

Returns:

dictionary Voting results, a dictionary in form of: {cluster label: assigned cell type}

Usage: DCS = DigitalCellSorter.DigitalCellSorter()

results = DCS.annotate(df_markers_expr, df_marker_cell_type)

Function `process()`: main function

DigitalCellSorter.**process**(*dataIsNormalized=False*, *cleanData=True*)

Process data before using any annotation of visualization functions

Parameters:

dataIsNormalized: boolean, Default False Whether DCS.df_expr is normalized or not

Returns: None

Usage: DCS = DigitalCellSorter.DigitalCellSorter()

DCS.process()

Function `visualize()`: make all default plots of to visualize results of function `process()`

DigitalCellSorter.**visualize**()

Aggregate of visualization tools of this class.

Parameters: None

Returns: None

Usage: DCS = DigitalCellSorter.DigitalCellSorter()

DCS.process()

DCS.visualize()

3.2 Extraction tools

Warning: Use these functions only after `process()`

References to DigitalCellSorter class:

`getExprOfGene`(gene[, analyzeBy])	Get expression of a gene.
`getExprOfCells`(cells)	Get expression of a set of cells.
`getCells`([celltype, clusterIndex, clusterName])	Get cell annotations in a form of pandas.Series
`getAnomalyScores`(trainingSet, testingSet[, . . .])	Function to get anomaly score of cells based on some reference set
`getNewMarkerGenes`([cluster, top, . . .])	Extract new marker genes based on the cluster annotations
`getIndexOfGoodQualityCells`([QCplotsSubDir])	Get index of sells that satisfy the QC criteria
`getCountsDataframe`(se1, se2[, tagForMissing])	Get a pandas.DataFrame with cross-counts (overlaps) between two pandas.Series

Function `getExprOfGene()`: Get expression of a gene

DigitalCellSorter.**getExprOfGene**(*gene*, *analyzeBy='cluster'*)

Get expression of a gene. Run this function only after function process()

Parameters:

cells: pandas.MultiIndex Index of cells of interest

analyzeBy: str, Default 'cluster' What level of lablels to include. Other possible options are 'label' and 'celltype'

Returns:

pandas.DataFrame With expression of the cells of interest

Usage: DCS = DigitalCellSorter.DigitalCellSorter()

DCS.process()

DCS.getExprOfGene('SDC1')

Function `getExprOfCells()`: Get expression of a set of cells

`DigitalCellSorter.`**`getExprOfCells`**`(`*cells*`)`

Get expression of a set of cells. Run this function only after function process()

Parameters:

cells: pandas.MultiIndex 2-level Index of cells of interest, must include levels 'batch' and 'cell'

Returns:

pandas.DataFrame With expression of the cells of interest

Usage: DCS = DigitalCellSorter.DigitalCellSorter()

DCS.process()

DCS.getExprOfCells(cells)

Function `getCells()`: get cells index by celltype, clusterIndex or clusterName

`DigitalCellSorter.`**`getCells`**`(`*celltype=None*, *clusterIndex=None*, *clusterName=None*`)`

Get cell annotations in a form of pandas.Series

Parameters:

celltype: str, Default None Cell type to extract

clusterIndex: int, Default None Cell type to extract

clusterName: str, Default None Cell type to extract

Returns:

pandas.MultiIndex Index of labelled cells

Usage: DCS = DigitalCellSorter.DigitalCellSorter()

DCS.process()

labels = DCS.getCells()

Function `getAnomalyScores()`: get anomaly score of cells based on some reference set

`DigitalCellSorter.`**`getAnomalyScores`**(*trainingSet*, *testingSet*, *printResults=False*)

Function to get anomaly score of cells based on some reference set

Parameters:

trainingSet: pandas.DataFrame With cells to trail isolation forest on

testingSet: pandas.DataFrame With cells to score

printResults: boolean, Default False Whether to print results

Returns:

1d numpy.array Anomaly score(s) of tested cell(s)

Usage: DCS = DigitalCellSorter.DigitalCellSorter()

cutoff = DCS.getAnomalyScores(df_expr.iloc[:, 5:], df_expr.iloc[:, :5])

Function `getNewMarkerGenes()`: extract new markers from the annotated clusters and produce plot of the new markers

`DigitalCellSorter.`**`getNewMarkerGenes`**(*cluster=None*, *top=100*, *zScoreCutoff=None*, *removeUnknown=False*, ***kwargs*)

Extract new marker genes based on the cluster annotations

Parameters:

cluster: int, Default None Cluster #, if provided genes of only this culster will be returned

top: int, Default 100 Upper bound for number of new markers per cell type

zScoreCutoff: float, Default 0.3 Lower bound for a marker z-score to be significant

removeUnknown: boolean, Default False Whether to remove type "Unknown"

Any parameters that function 'makePlotOfNewMarkers' can accept

Returns: None

Usage: DCS = DigitalCellSorter.DigitalCellSorter()

DCS.extractNewMarkerGenes()

Function `getIndexOfGoodQualityCells()`: Get index of sells that satisfy the QC criteria

`DigitalCellSorter.`**`getIndexOfGoodQualityCells`**(*QCplotsSubDir='QC_plots'*, ***kwargs*)

Get index of sells that satisfy the QC criteria

Parameters:

count_depth_cutoff: float, Default 0.5 Fraction of median to take as count depth cutoff

number_of_genes_cutoff: float, Default 0.5 Fraction of median to take as number of genes cutoff

mitochondrial_genes_cutoff: float, Default 3.0 The cutoff is median + standard_deviation * this_parameter

Any parameters that function 'makeQualityControlHistogramPlot' can accept

Returns:

pandas.Index Index of cells

Usage: DCS = DigitalCellSorter.DigitalCellSorter()

index = DCS.getIndexOfGoodQualityCells()

Function `getCountsDataframe()`: Get a pandas.DataFrame with cross-counts (overlaps) between two pandas.Series

`DigitalCellSorter.`**`getCountsDataframe`**(*se1*, *se2*, *tagForMissing='N/A'*)

Get a pandas.DataFrame with cross-counts (overlaps) between two pandas.Series

Parameters:

se1: pandas.Series Series with the first set of items

se2: pandas.Series Series with the second set of items

tagForMissing: str, Default 'N/A' Label to assign to non-overlapping items

Returns:

pandas.DataFrame Contains counts

Usage: DCS = DigitalCellSorter.DigitalCellSorter()

df = DCS.getCountsDataframe(se1, se2)

3.3 Visualization tools

> **Warning:** Use these functions only after `process()`

References to DigitalCellSorter class:

makeProjectionPlotAnnotated(**kwargs)	Produce projection plot colored by cell types

Continued on next page

Table 3 – continued from previous page

makeProjectionPlotByBatches(**kwargs)	Produce projection plot colored by batches
makeProjectionPlotByClusters(**kwargs)	Produce projection plot colored by clusters
makeProjectionPlotsQualityControl(**kwargs)	Produce Quality Control projection plots
makeMarkerSubplots(**kwargs)	Produce subplots on each marker and its expression on all clusters
makeAnomalyScoresPlot([cells, suffix, noPlot])	Make anomaly scores plot
makeIndividualGeneTtestPlot(gene[, analyzeBy])	Produce individual gene t-test plot of the two-tailed p-value.
makeIndividualGeneExpressionPlot(genes, **kwargs)	Produce individual gene expression plot on a 2D layout

References to VisualizationFunctions class:

makeQualityControlHistogramPlot(*args, **kwargs)
makeHistogramNullDistributionPlot(*args, …)
makeAnnotationResultsMatrixPlot(*args, **kwargs)
makeMarkerExpressionPlot(*args, **kwargs)
makeStackedBarplot(*args, **kwargs)
makeSankeyDiagram(*args, **kwargs)

Function `makeProjectionPlotAnnotated()`: Produce t-SNE plot colored by cell types

`DigitalCellSorter.`**`makeProjectionPlotAnnotated`**`(`***kwargs*`)`

Produce projection plot colored by cell types

Parameters: Any parameters that function 'makeProjectionPlot' can accept

Returns: None

Usage: DCS = DigitalCellSorter.DigitalCellSorter()

DCS.process()

DCS.makeProjectionPlotAnnotated()

Example output:

Function `makeProjectionPlotByBatches()`: Produce t-SNE plot colored by batches

`DigitalCellSorter.`**`makeProjectionPlotByBatches`**`(`***kwargs*`)`

Produce projection plot colored by batches

Parameters: Any parameters that function 'makeProjectionPlot' can accept

Returns: None

Usage: DCS = DigitalCellSorter.DigitalCellSorter()

DCS.process()

DCS.makeProjectionPlotByBatches()

Example output:

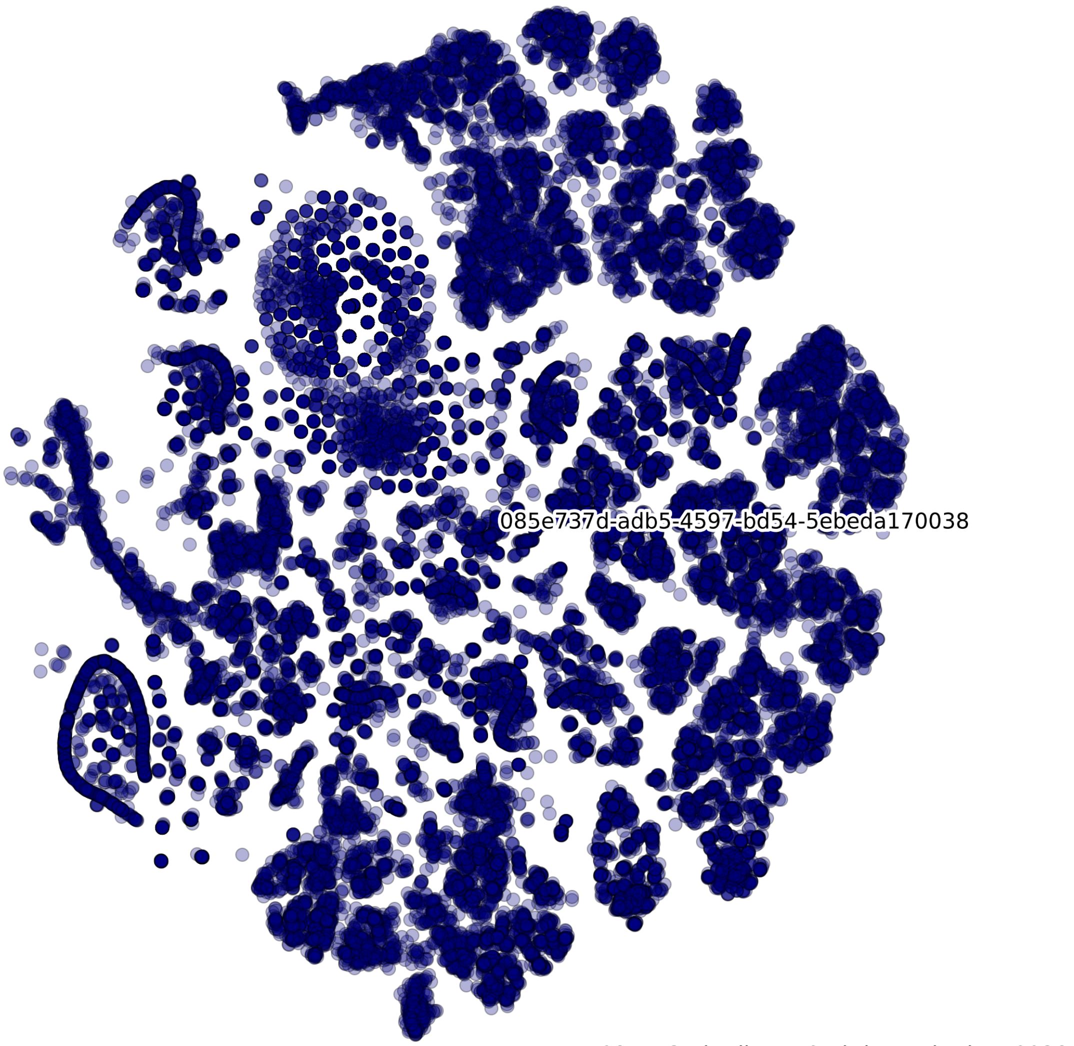

Function `makeProjectionPlotByClusters()`: Produce t-SNE plot colored by clusters

`DigitalCellSorter.`**`makeProjectionPlotByClusters`**`(`***kwargs*`)`

Produce projection plot colored by clusters

Parameters: Any parameters that function 'makeProjectionPlot' can accept

Returns: None

Usage: DCS = DigitalCellSorter.DigitalCellSorter()

DCS.process()

DCS.makeProjectionPlotByClusters()

Example output:

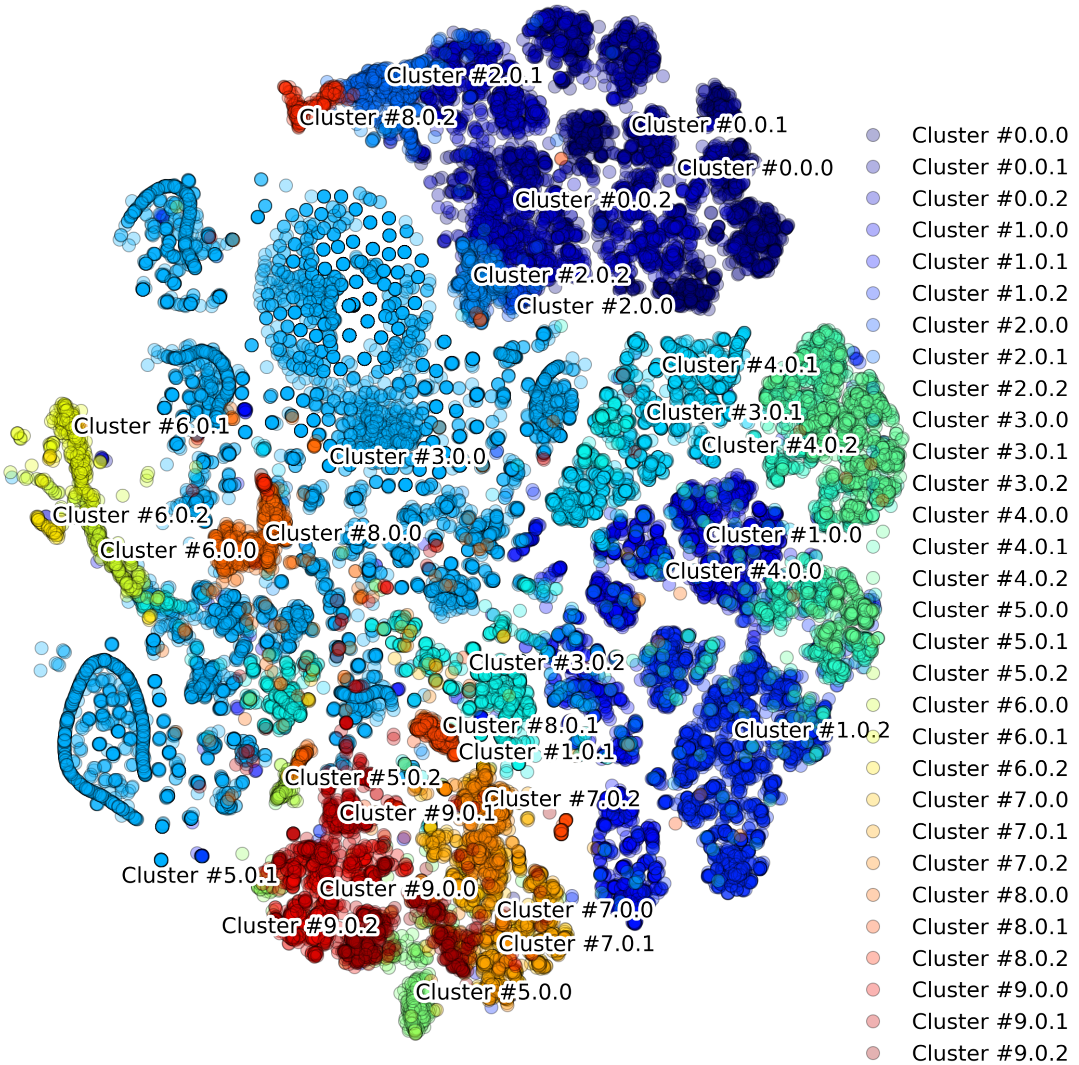

Function `makeQualityControlHistogramPlot()`: Produce Quality Control histogram plots

`DigitalCellSorter.`**`makeQualityControlHistogramPlot`**`(`**args*, ***kwargs*`)`

Example output:

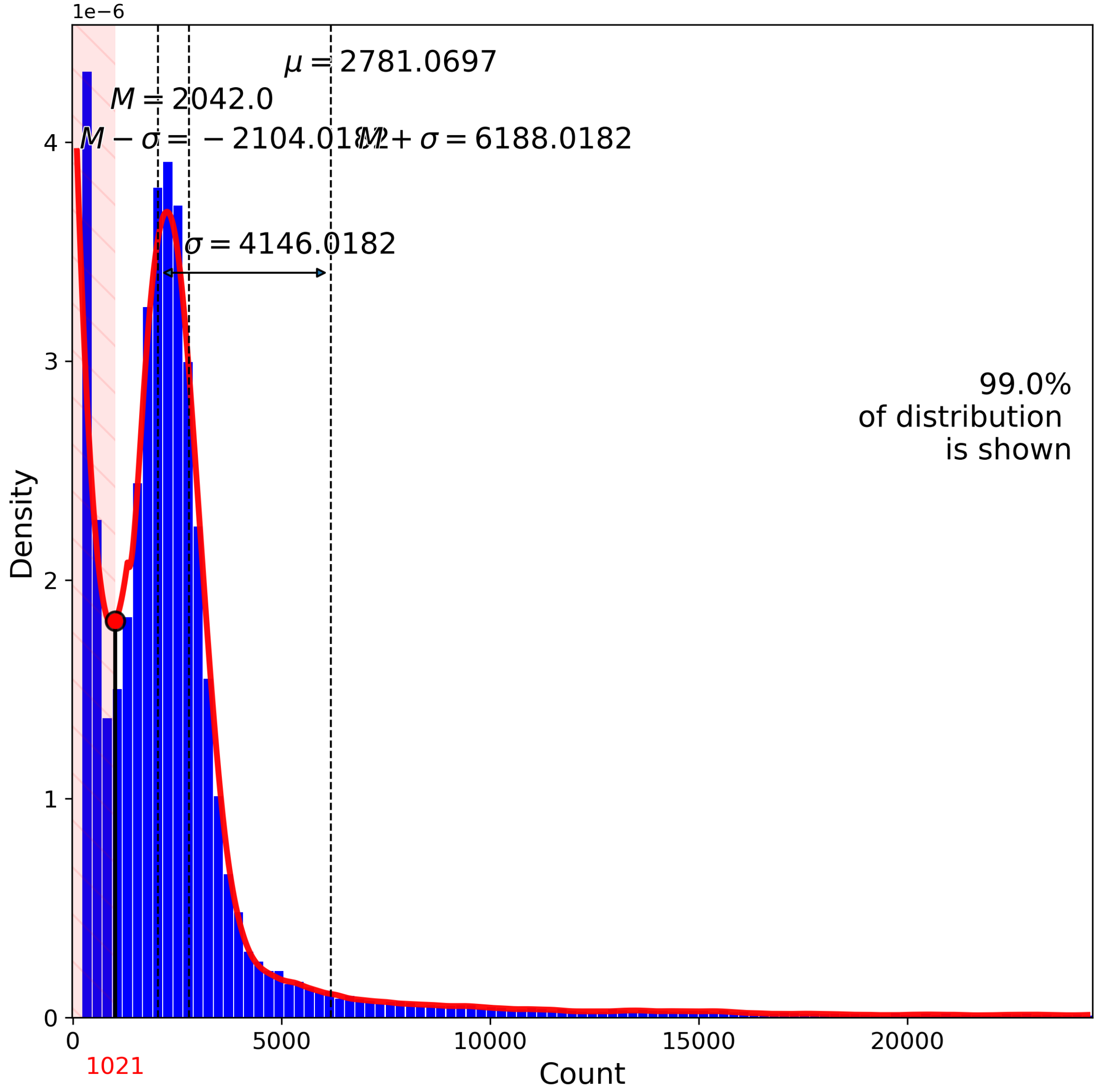

Function `makeProjectionPlotsQualityControl()`: Produce Quality Control t-SNE plots

`DigitalCellSorter.`**`makeProjectionPlotsQualityControl`**`(`***kwargs*`)`

Produce Quality Control projection plots

Parameters: Any parameters that function 'makeProjectionPlot' can accept

Returns: None

Usage: DCS = DigitalCellSorter.DigitalCellSorter()

DCS.process()

DCS.makeProjectionPlotsQualityControl()

Example output:

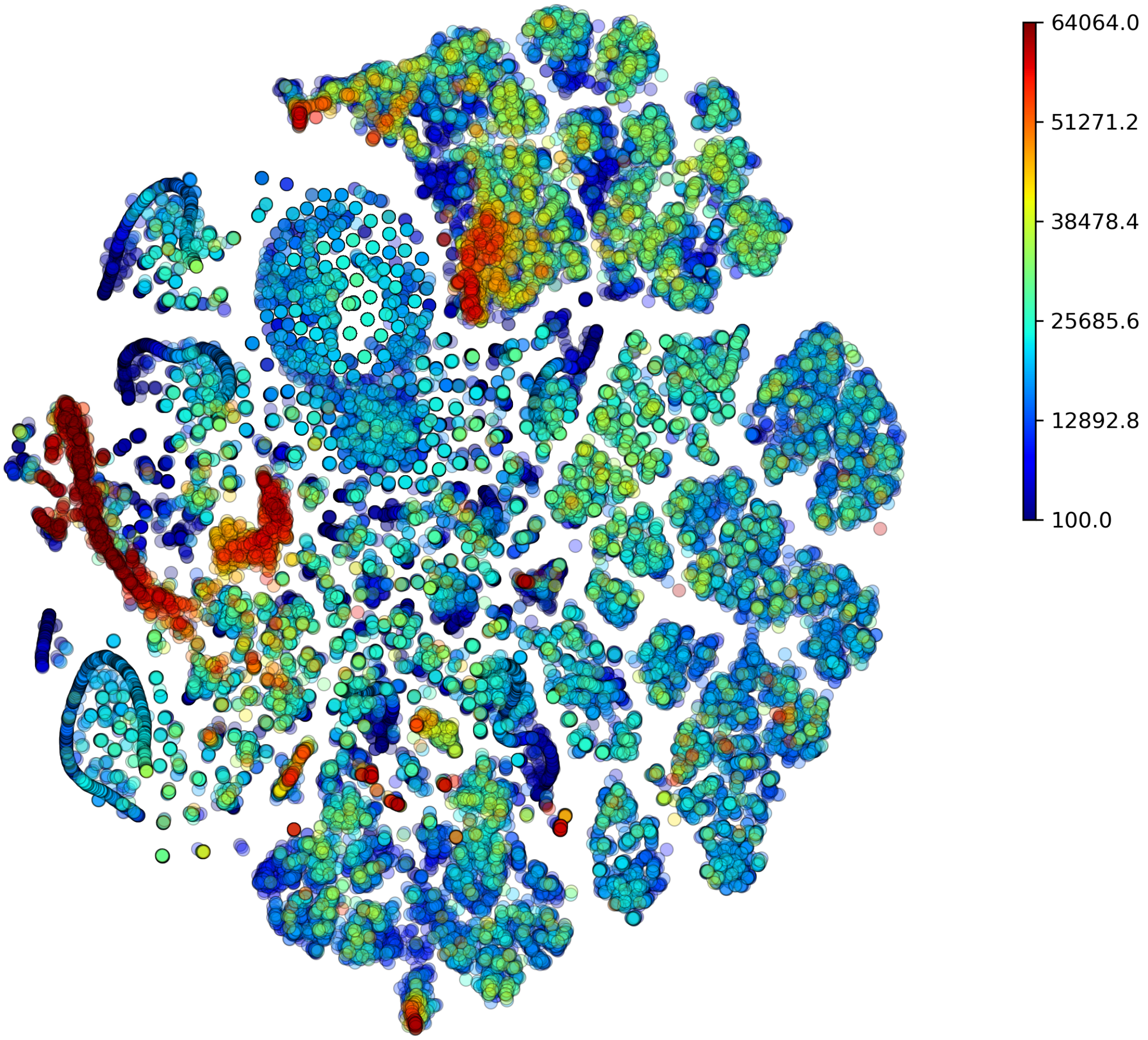

Function `makeMarkerSubplots()`: Produce subplots on each marker and its expression on all clusters

`DigitalCellSorter.`**`makeMarkerSubplots`**`(**kwargs)`

Produce subplots on each marker and its expression on all clusters

Parameters: Any parameters that function 'internalMakeMarkerSubplots' can accept

Returns: None

Usage: DCS = DigitalCellSorter.DigitalCellSorter()

DCS.process()

DCS.makeMarkerSubplots()

Example output:

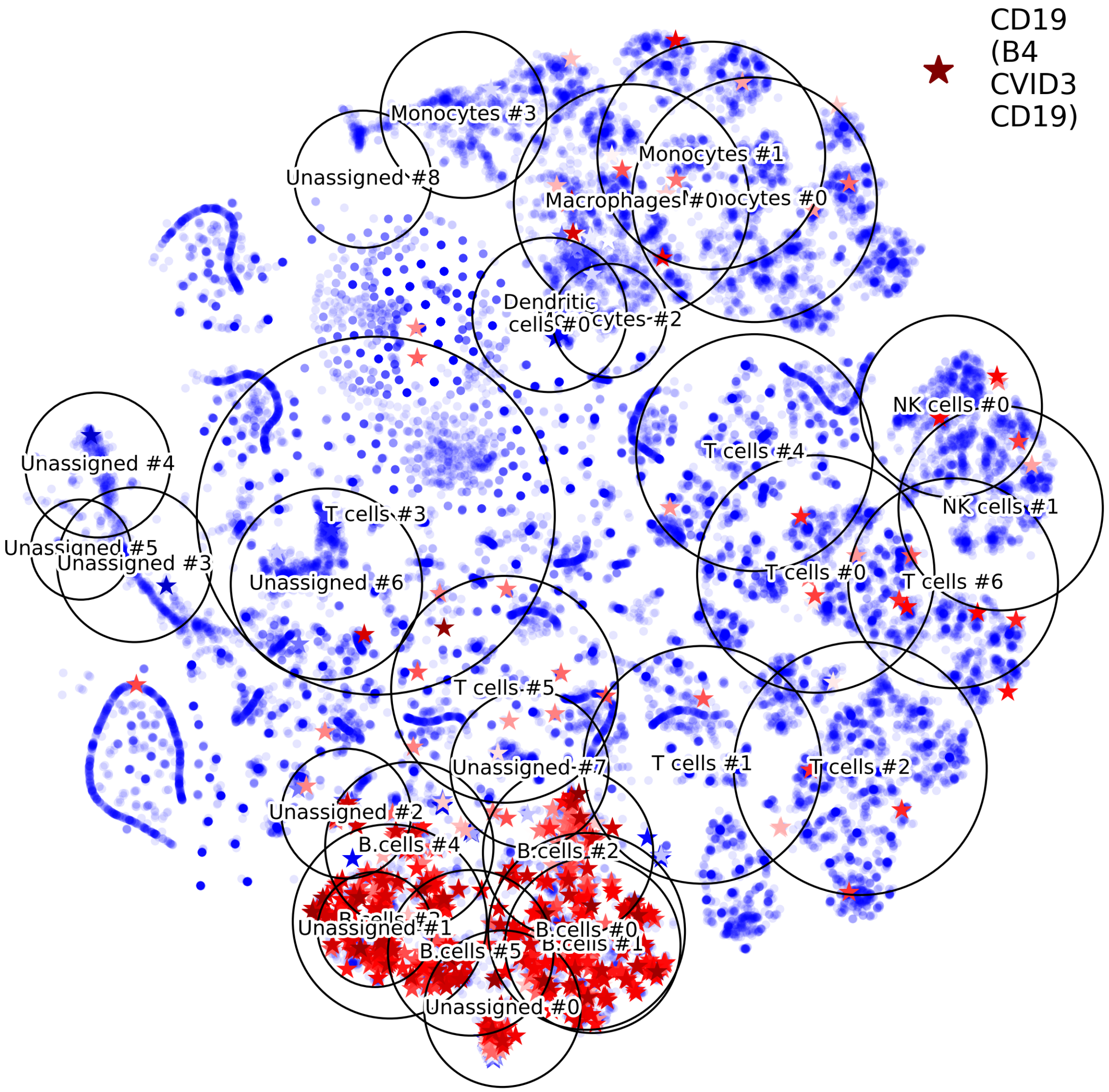

Function `makeAnomalyScoresPlot()`: Make anomaly scores plot

`DigitalCellSorter.`**`makeAnomalyScoresPlot`**(*cells='All'*, *suffix=''*, *noPlot=False*, ***kwargs*)

Make anomaly scores plot

Parameters:

cells: pandas.MultiIndex, Default 'All' Index of cells of interest

Any parameters that function 'makeProjectionPlot' can accept

Returns: None

Usage: DCS = DigitalCellSorter.DigitalCellSorter()

DCS.process()

cells = DCS.getCells(celltype='T cell')

DCS.makeAnomalyScoresPlot(cells)

Example output:

Function `makeIndividualGeneTtestPlot()`: Produce individual gene t-test plot of the two-tailed p-value

`DigitalCellSorter.`**`makeIndividualGeneTtestPlot`**(*gene*, *analyzeBy='label'*, ***kwargs*)

Produce individual gene t-test plot of the two-tailed p-value.

Parameters:

gene: str Name of gene of interest

analyzeBy: str, Default 'label' What level of lablels to include. Other possible options are 'label' and 'celltype'

Any parameters that function 'makeTtestPlot' can accept

Returns: None

Usage: DCS = DigitalCellSorter.DigitalCellSorter()

DCS.makeIndividualGeneTtestPlot('SDC1')

Example output:

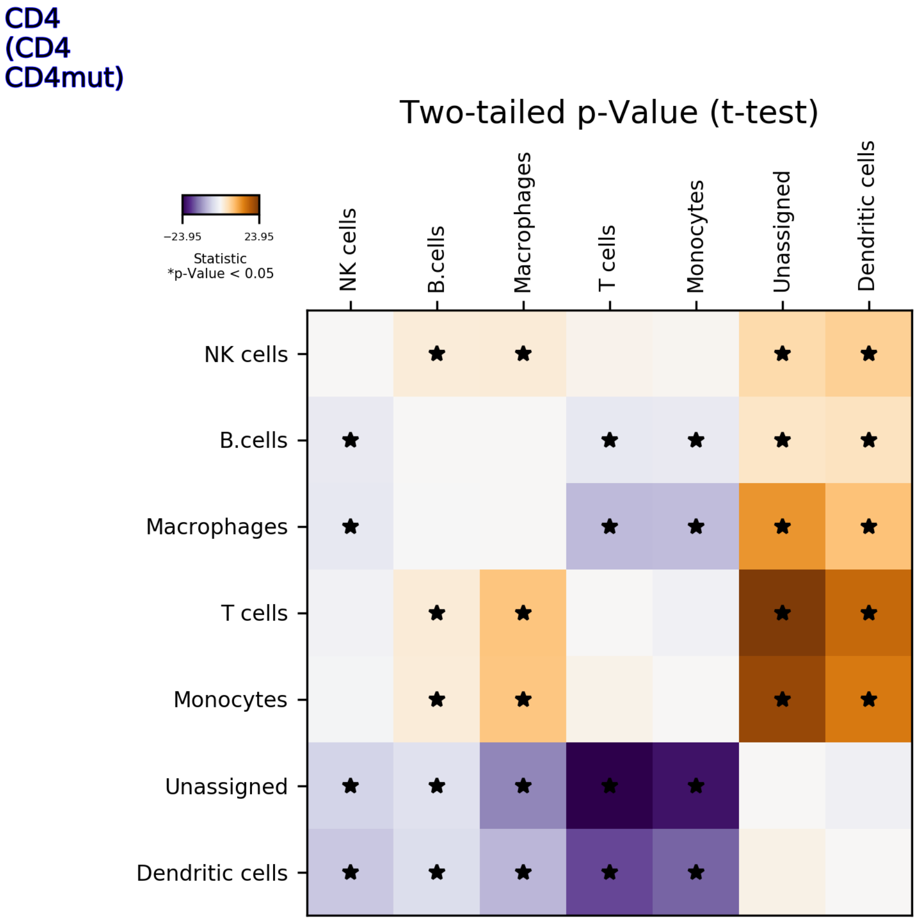

Function `makeIndividualGeneExpressionPlot()`: Produce individual gene expression plot on a 2D layout

`DigitalCellSorter.`**`makeIndividualGeneExpressionPlot`**(*genes*, ***kwargs*)

Produce individual gene expression plot on a 2D layout

Parameters:

gene: str, or list-like Name of gene of interest. E.g. ‘CD4, CD33’, ‘PECAM1’, [‘CD4’, ‘CD33’]

hideClusterLabels: boolean, Default False Whether to hide the clusters labels

outlineClusters: boolean, Default True Whether to outline the clusters with circles

Any parameters that function ‘internalMakeMarkerSubplots’ can accept

Returns: None

Usage: DCS = DigitalCellSorter.DigitalCellSorter()

DCS.makeIndividualGeneExpressionPlot(‘CD4’)

Example output:

Function `makeHistogramNullDistributionPlot()`: Produce histogram plot of the voting null distributions

`DigitalCellSorter.`**`makeHistogramNullDistributionPlot`**`(`**args*, ***kwargs*`)`

Example output:

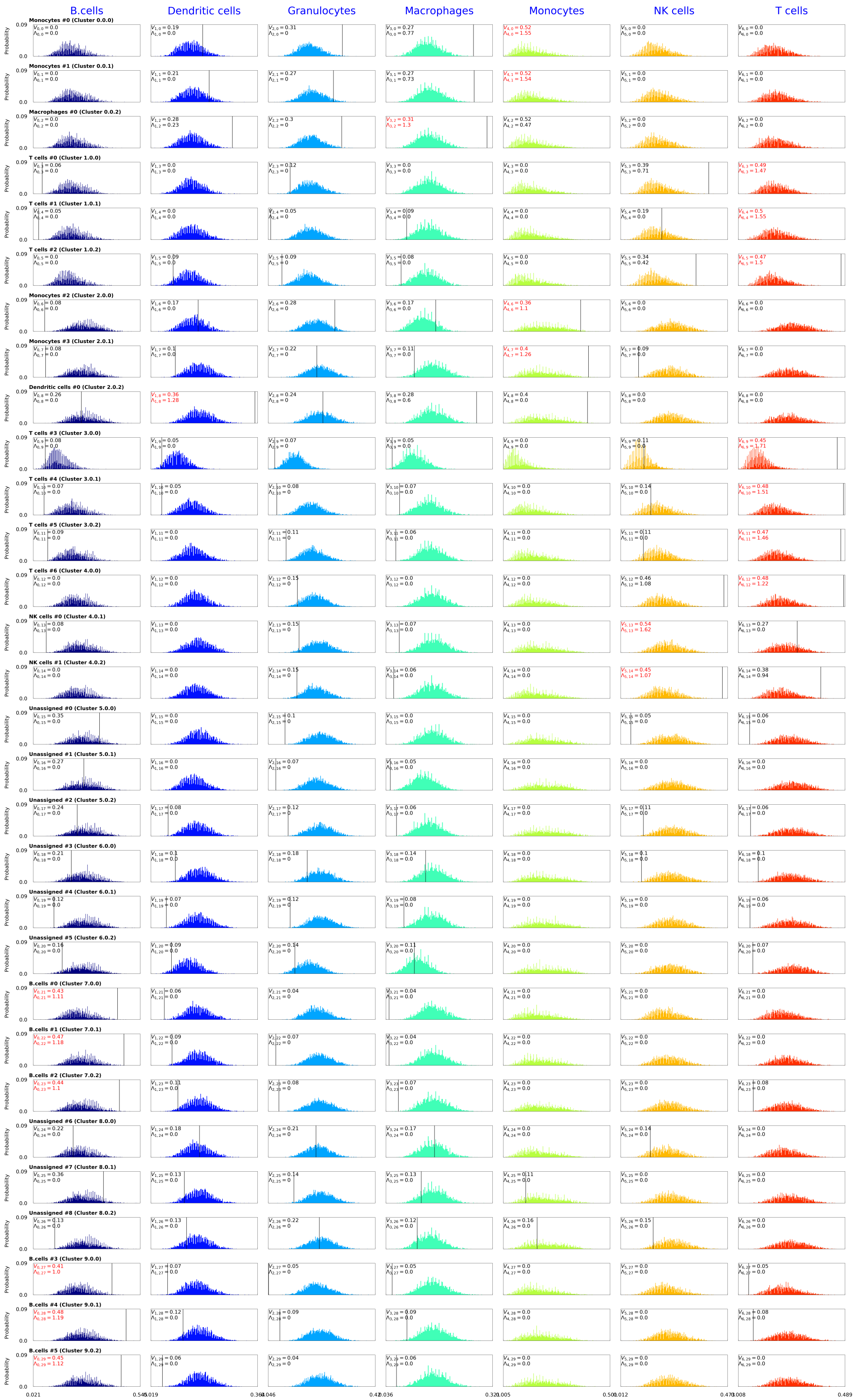
B.cells
Dendritic cells
Granulocytes
Macrophages
Monocytes
NK cells
T cells

Function `makeAnnotationResultsMatrixPlot()`: Produce voting results voting matrix plot

`DigitalCellSorter.`**`makeAnnotationResultsMatrixPlot`**`(`**args*, ***kwargs*`)`

Example output:

Function `makeMarkerExpressionPlot()`: Produce image on marker genes and their expression on all clusters

`DigitalCellSorter.`**`makeMarkerExpressionPlot`**`(`**args*, ***kwargs*`)`

Example output:

Function `makeStackedBarplot()`: Produce stacked barplot with cell fractions

`DigitalCellSorter.`**`makeStackedBarplot`**`(`**args*, ***kwargs*`)`

Example output:

Function `makeSankeyDiagram()`: Make a Sankey diagram, also known as 'river plot' with two groups of nodes

`DigitalCellSorter.`**`makeSankeyDiagram`**`(`**args*, ***kwargs*`)`

Example output:

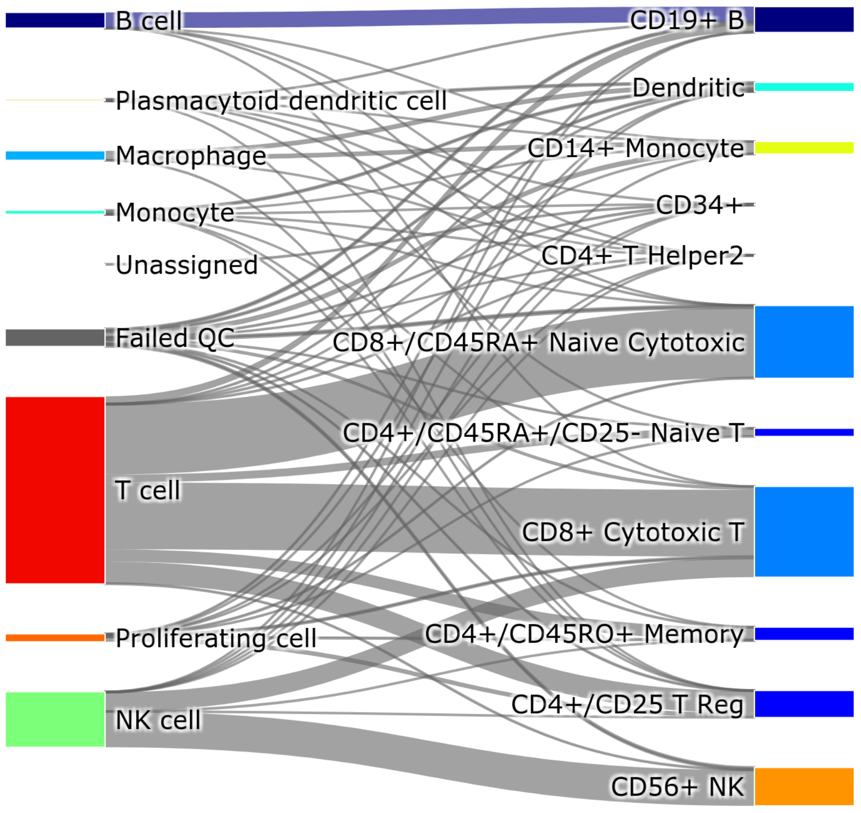
B cell
Plasmacytoid dendritic cell
Macrophage
Monocyte
Unassigned
Failed QC
T cell
Proliferating cell
NK cell
CD19+ B
Dendritic
CD14+ Monocyte
CD34+
CD4+ T Helper2
CD8+/CD45RA+ Naive Cytotoxic
CD4+/CD45RA+/CD25- Naive T
CD8+ Cytotoxic T
CD4+/CD45RO+ Memory
CD4+/CD25 T Reg
CD56+ NK

CHAPTER

FOUR

CORE CLASS

Submodule core

Description of the package functionality

The main class of DigitalCellSorter. The class includes tools for:

1. **Pre-preprocessing** of single cell RNA sequencing data
2. **Quality control**
3. **Batch effects correction**
4. **Cells anomaly score evaluation**
5. **Dimensionality reduction**
6. **Clustering**
7. **Annotation of cell types**
8. **Vizualization**
9. **Post-processing**

class DigitalCellSorter(*df_expr=None*, *dataName='dataName'*, *species='Human'*, *geneNamesType='alias'*, *geneListFileName=None*, *mitochondrialGenes=None*, *sigmaOverMeanSigma=0.01*, *nClusters=10*, *nFineClusters=3*, *doFineClustering=True*, *splitFineClusters=False*, *subSplitSize=100*, *medianScaleFactor=10000*, *minSizeForFineClustering=50*, *clusteringFunction=<class 'sklearn.cluster._agglomerative.AgglomerativeClustering'>*, *nComponentsPCA=200*, *nSamples_pDCS=3000*, *nSamples_Hopfield=200*, *saveDir=''*, *makeMarkerSubplots=False*, *availableCPUsCount=1*, *zScoreCutoff=0.3*, *subclusteringName=None*, *doQualityControl=True*, *doBatchCorrection=False*, *makePlots=True*, *useUnderlyingNetwork=True*, *minimumNumberOfMarkersPerCelltype=10*, *nameForUnknown='Unassigned'*, *nameForLowQC='Failed QC'*, *matplotlibMode='Agg'*, *countDepthCutoffQC=0.5*, *numberOfGenesCutoffQC=0.5*, *mitochondrialGenesCutoffQC=1.5*, *excludedFromQC=None*, *countDepthPrecutQC=500*, *numberOfGenesPrecutQC=250*, *precutQC=False*, *minSubclusterSize=25*, *thresholdForUnknown_pDCS=0.0*, *thresholdForUnknown_ratio=0.0*, *thresholdForUnknown_Hopfield=0.0*, *thresholdForUnknown=0.2*, *layout='TSNE'*, *safePlotting=True*, *HopfieldTemperature=0.1*, *annotationMethod='ratio-pDCS-Hopfield'*, *useNegativeMarkers=True*, *removeLowQualityScores=True*, *updateConversionDictFile=True*, *verbose=1*)

Bases: `DigitalCellSorter.VisualizationFunctions.VisualizationFunctions`

Class of Digital Cell Sorter with methods for processing single cell RNA-seq data. Includes analyses and visualization tools.

Parameters:

df_expr: pandas.DataFrame, Defauld None Gene expression in a form of a table, where genes are rows, and cells/batches are columns

dataName: str, Default 'dataName' Name used in output files

geneNamesType: str, Default 'alias' Input gene name convention

geneListFileName: str, Default None Name of the marker genes file

mitochondrialGenes: list, Default None List of mitochondrial genes to use in quality control

sigmaOverMeanSigma: float, Default 0.1 Threshold to consider a gene constant

nClusters: int, Default 10 Number of clusters

nFineClusters: int, Default 3 Number of fine clusters to determine with Spectral Co-clustering routine. This option is ignored is doFineClustering is False.

doFineClustering: boolean, Default True Whether to do fine clustering or not

minSizeForFineClustering: int, Default 50 Minimum number of cells required to do fine clustering of a cluster. This option is ignored is doFineClustering is False.

clusteringFunction: function, Default AgglomerativeClustering Clustering function to use. Other options: KMeans, {k_neighbors:40}, etc. Note: the function should have .fit method and same input and output. For Network-based clustering pass a dictionary {'k_neighbors':40, metric:'euclidean', 'clusterExpression':True}, this way the best number of clusters will be determined automatically

nComponentsPCA: int, Default 200 Number of pca components

nSamples_pDCS: int, Default 3000 Number of random samples in distribution for pDCS annotation method

nSamples_Hopfield: int, Default 500 Number of repetitions for Hopfield annotation method

saveDir: str, Default os.path.join('') Directory for output files

makeMarkerSubplots: boolean, Default False Whether to make subplots on markers

makePlots: boolean, Default True Whether to make all major plots

availableCPUsCount: int, Default min(12, os.cpu_count()) Number of CPUs used in pDCS method

zScoreCutoff: float, Default 0.3 Z-Score cutoff when setting expression of a cluster as significant

thresholdForUnknown: float, Default 0.3 Threshold when assigning label "Unknown". This option is used only with a combination of 2 or more annotation methods

thresholdForUnknown_pDCS: float, Default 0.1 Threshold when assigning label "Unknown" in pDCS method

thresholdForUnknown_ratio: float, Default 0.1 Threshold when assigning label "Unknown" in ratio method

thresholdForUnknown_Hopfield: float, Default 0.1 Threshold when assigning label "Unknown" in Hopfield method

annotationMethod: str, Default 'ratio-pDCS-Hopfield'

Metod to use for annotation of cell types to clusters. Options are: 'pDCS': main DCS voting scheme with null testing

'ratio': simple voting score

'Hopfield': Hopfield Network classifier

'pDCS-ratio': 'pDCS' adjusted with 'ratio'

'pDCS-Hopfield': 'pDCS' adjusted with 'Hopfield'

'ratio-Hopfield': 'ratio' adjusted with 'Hopfield'

'pDCS-ratio-Hopfield': 'pDCS' adjusted with 'ratio' and 'Hopfield'

subclusteringName: str, Default None Parameter used in for certain labels on plots

doQualityControl: boolean, Default True Whether to remove low quality cells

doBatchCorrection: boolean, Default False Whether to correct data for batches

minimumNumberOfMarkersPerCelltype: int, Default 10 Minimum number of markers per cell type to keep that cell type in annotation options

nameForUnknown: str, Default 'Unassigned' Name to use for clusters where label assignment yielded uncertain results

nameForLowQC: str, Default 'Failed QC' Name to use for cell that do not pass quality control

layout: str, Default 'TSNE'

Projection layout used in visualization. Options are: 'TSNE': t-SNE layout L.J.P. van der Maaten. Accelerating t-SNE using Tree-Based Algorithms. Journal of Machine Learning Research 15(Oct):3221-3245, 2014.

'PCA': use two largest principal components

'UMAP': use uniform manifold approximation, McInnes, L., Healy, J., UMAP: Uniform Manifold Approximation and Projection for Dimension Reduction, ArXiv e-prints 1802.03426, 2018

'PHATE': use potential of heat diffusion for affinity-based transition embedding, Moon, K.R., van Dijk, D., Wang, Z. et al. Visualizing structure and transitions in high-dimensional biological data. Nat Biotechnol 37, 1482–1492 (2019).

Usage: DCS = DigitalCellSorter.DigitalCellSorter()

df_data = DCS.Clean(df_data)

Methods:

KeyInFile(key, file)	Check is a key exists in a HDF file.
alignSeries(se1, se2, tagForMissing)	Align two pandas.Series
annotate([mapNonexpressedCelltypes])	Produce cluster voting results, annotate cell types, and update marker expression with cell type labels
annotateWith_Hopfield_Scheme(. . .)	Produce cluster annotation results
annotateWith_pDCS_Scheme(df_markers_expr, . . .)	Produce cluster annotation results
annotateWith_ratio_Scheme(df_markers_expr, . . .)	Produce cluster annotation results
batchEffectCorrection([method])	Batch effect correction.
calculateQCmeasures()	Calculate Quality Control (QC) measures
calculateV(args)	Calculate the voting scores (celltypes by clusters)
clean()	Clean pandas.DataFrame: validate index, remove index duplicates, replace missing with zeros, remove all-zero rows and columns
cluster()	Cluster PCA-reduced data into a desired number of clusters
convert([nameFrom, nameTo])	Convert index to hugo names, if any names in the index are duplicated, remove duplicates
convertColormap(colormap)	Convert colormap from the form (1.,1.,1.,1.) to 'rgba(255,255,255,1.)'
createReverseDictionary(inputDictionary)	Efficient way to create a reverse dictionary from a dictionary.
getAnomalyScores(trainingSet, testingSet[, . . .])	Function to get anomaly score of cells based on some reference set
getCells([celltype, clusterIndex, clusterName])	Get cell annotations in a form of pandas.Series
getCountsDataframe(se1, se2[, tagForMissing])	Get a pandas.DataFrame with cross-counts (overlaps) between two pandas.Series
getExprOfCells(cells)	Get expression of a set of cells.
getExprOfGene(gene[, analyzeBy])	Get expression of a gene.
getHugoName(gene[, printAliases])	Get gene hugo name(s).
getIndexOfGoodQualityCells([QCplotsSubDir])	Get index of sells that satisfy the QC criteria
getNewMarkerGenes([cluster, top, . . .])	Extract new marker genes based on the cluster annotations
getQualityControlCutoff(se, cutoff[, . . .])	Function to calculate QC quality cutoff
getSubnetworkOfPCN(subnetworkGenes[, . . .])	Extract subnetwork of PCN network
loadAnnotatedLabels([detailed, . . .])	Load cell annotations resulted from function 'annotate'
loadExpressionData()	Load processed expression data from the internal HDF storage.
makeAnomalyScoresPlot([cells, suffix, noPlot])	Make anomaly scores plot
makeHopfieldLandscapePlot([. . .])	Make and plot Hopfield landscape
makeIndividualGeneExpressionPlot(genes, **kwargs)	Produce individual gene expression plot on a 2D layout

Continued on next page

Table 1 – continued from previous page

makeIndividualGeneTtestPlot(gene[, analyzeBy])	Produce individual gene t-test plot of the two-tailed p-value.
makeMarkerSubplots(**kwargs)	Produce subplots on each marker and its expression on all clusters
makeProjectionPlotAnnotated(**kwargs)	Produce projection plot colored by cell types
makeProjectionPlotByBatches(**kwargs)	Produce projection plot colored by batches
makeProjectionPlotByClusters(**kwargs)	Produce projection plot colored by clusters
makeProjectionPlotsQualityControl(**kwargs)	Produce Quality Control projection plots
mergeIndexDuplicates(df_expr[, method, …])	Merge index duplicates
normalize([median])	Normalize pandas.DataFrame: rescale all cells, log-transform data, remove constant genes, sort index
prepare(obj)	Prepare pandas.DataFrame for input to function process() If input is pd.DataFrame validate the input whether it has correct structure.
prepareMarkers([expressedGenes, …])	Get dictionary of markers for each cell types.
process([dataIsNormalized, cleanData])	Process data before using any annotation of visualization functions
project([PCAonly, do_fast_tsne])	Project pandas.DataFrame to lower dimensions
propagateHopfield([sigma, xi, T, tmax, …])	Function is used internally to propagate Hopfield network over a set number of time steps
qualityControl(**kwargs)	Remove low quality cells
readMarkerFile([mergeFunction, mergeCutoff])	Read markers file, prepare markers
recordAnnotationResults(df_marker_cell_type, …)	Record cell type annotation results to spreadsheets.
recordExpressionData()	Record expression data from the internal HDF storage.
visualize()	Aggregate of visualization tools of this class.
zScoreOfSeries(se)	Calculate z-score of pandas.Series and modify the Series in place

Attributes:

df_expr
fileHDFpath
geneListFileName
saveDir

property saveDir

property fileHDFpath

property df_expr

property geneListFileName

prepare(*obj*)

Prepare pandas.DataFrame for input to function process() If input is pd.DataFrame validate the input whether it has correct structure.

Parameters:

obj: str, pandas.DataFrame, pandas.Series Expression data in a form of pandas.DataFrame, pan-

das.Series, or name and path to a csv file with data

Returns: None

Usage: DCS = DigitalCellSorter.DigitalCellSorter()

dDCS.preapre('data.csv')

convert (*nameFrom=None*, *nameTo=None*, ***kwargs*)

Convert index to hugo names, if any names in the index are duplicated, remove duplicates

Parameters:

nameFrom: str, Default 'alias' Gene name type to convert from

nameTo: str, Default 'hugo' Gene name type to convert to

Any parameters that function 'mergeIndexDuplicates' can accept

Returns: None

Usage: DCS = DigitalCellSorter.DigitalCellSorter()

DCS.convertIndex()

clean ()

Clean pandas.DataFrame: validate index, remove index duplicates, replace missing with zeros, remove all-zero rows and columns

Parameters: None

Returns: None

Usage: DCS = DigitalCellSorter.DigitalCellSorter()

DCS.clean()

normalize (*median=None*)

Normalize pandas.DataFrame: rescale all cells, log-transform data, remove constant genes, sort index

Parameters:

median: float, Default None Scale factor, if not provided will be computed as median across all cells in data

Returns: None

Usage: DCS = DigitalCellSorter.DigitalCellSorter()

DCS.normalize()

project (*PCAonly=False*, *do_fast_tsne=True*)

Project pandas.DataFrame to lower dimensions

Parameters:

PCAonly: boolean, Default False Perform Principal component analysis only

do_fast_tsne: boolean, Default True Do FI-tSNE instead of "exact" tSNE This option is ignored if layout is not 'TSNE'

Returns:

tuple Processed data

Usage: DCS = DigitalCellSorter.DigitalCellSorter()

xPCA, PCs, tSNE = DCS.project()

cluster()

Cluster PCA-reduced data into a desired number of clusters

Parameters: None

Returns: None

Usage: DCS = DigitalCellSorter.DigitalCellSorter()

DCS.cluster()

annotate(*mapNonexpressedCelltypes=True*)

Produce cluster voting results, annotate cell types, and update marker expression with cell type labels

Parameters:

mapNonexpressedCelltypes: boolean, Default True If True then cell types coloring will be consistent across all datasets, regardless what cell types are annotated in all datasets for a given input marker list file.

Returns:

dictionary Voting results, a dictionary in form of: {cluster label: assigned cell type}

Usage: DCS = DigitalCellSorter.DigitalCellSorter()

results = DCS.annotate(df_markers_expr, df_marker_cell_type)

process(*dataIsNormalized=False*, *cleanData=True*)

Process data before using any annotation of visualization functions

Parameters:

dataIsNormalized: boolean, Default False Whether DCS.df_expr is normalized or not

Returns: None

Usage: DCS = DigitalCellSorter.DigitalCellSorter()

DCS.process()

visualize()

Aggregate of visualization tools of this class.

Parameters: None

Returns: None

Usage: DCS = DigitalCellSorter.DigitalCellSorter()

DCS.process()

DCS.visualize()

makeProjectionPlotAnnotated(***kwargs*)

Produce projection plot colored by cell types

Parameters: Any parameters that function 'makeProjectionPlot' can accept

Returns: None

Usage: DCS = DigitalCellSorter.DigitalCellSorter()

DCS.process()

DCS.makeProjectionPlotAnnotated()

makeProjectionPlotByBatches(***kwargs*)

Produce projection plot colored by batches

Parameters: Any parameters that function 'makeProjectionPlot' can accept

Returns: None

Usage: DCS = DigitalCellSorter.DigitalCellSorter()

DCS.process()

DCS.makeProjectionPlotByBatches()

makeProjectionPlotByClusters (***kwargs*)
Produce projection plot colored by clusters

Parameters: Any parameters that function 'makeProjectionPlot' can accept

Returns: None

Usage: DCS = DigitalCellSorter.DigitalCellSorter()

DCS.process()

DCS.makeProjectionPlotByClusters()

makeProjectionPlotsQualityControl (***kwargs*)
Produce Quality Control projection plots

Parameters: Any parameters that function 'makeProjectionPlot' can accept

Returns: None

Usage: DCS = DigitalCellSorter.DigitalCellSorter()

DCS.process()

DCS.makeProjectionPlotsQualityControl()

makeMarkerSubplots (***kwargs*)
Produce subplots on each marker and its expression on all clusters

Parameters: Any parameters that function 'internalMakeMarkerSubplots' can accept

Returns: None

Usage: DCS = DigitalCellSorter.DigitalCellSorter()

DCS.process()

DCS.makeMarkerSubplots()

makeAnomalyScoresPlot (*cells='All'*, *suffix=''*, *noPlot=False*, ***kwargs*)
Make anomaly scores plot

Parameters:

cells: pandas.MultiIndex, Default 'All' Index of cells of interest

Any parameters that function 'makeProjectionPlot' can accept

Returns: None

Usage: DCS = DigitalCellSorter.DigitalCellSorter()

DCS.process()

cells = DCS.getCells(celltype='T cell')

DCS.makeAnomalyScoresPlot(cells)

makeIndividualGeneTtestPlot (*gene*, *analyzeBy='label'*, ***kwargs*)
Produce individual gene t-test plot of the two-tailed p-value.

Parameters:

gene: str Name of gene of interest

analyzeBy: str, Default 'label' What level of lablels to include. Other possible options are 'label' and 'celltype'

Any parameters that function 'makeTtestPlot' can accept

Returns: None

Usage: DCS = DigitalCellSorter.DigitalCellSorter()

DCS.makeIndividualGeneTtestPlot('SDC1')

makeIndividualGeneExpressionPlot (*genes*, ***kwargs*)
Produce individual gene expression plot on a 2D layout

Parameters:

gene: str, or list-like Name of gene of interest. E.g. 'CD4, CD33', 'PECAM1', ['CD4', 'CD33']

hideClusterLabels: boolean, Default False Whether to hide the clusters labels

outlineClusters: boolean, Default True Whether to outline the clusters with circles

Any parameters that function 'internalMakeMarkerSubplots' can accept

Returns: None

Usage: DCS = DigitalCellSorter.DigitalCellSorter()

DCS.makeIndividualGeneExpressionPlot('CD4')

makeHopfieldLandscapePlot (*meshSamplingRate=1000*, *plot3D=True*, *reuseData=False*, ***kwargs*)
Make and plot Hopfield landscape

Parameters:

meshSamplingRate: int, Default 1000 Defines quality of sampling around attractor states

plot3D: boolean, Default False Whether to plot 2D or 3D figure

reuseData: boolean, Default False Whether to attempt using precalculated data.

Any parameters that function 'HopfieldLandscapePlot' or 'HopfieldLandscapePlot3D' can accept

Returns: None

Usage: DCS = DigitalCellSorter.DigitalCellSorter() DCS.makeHopfieldLandscapePlot()

getAnomalyScores (*trainingSet*, *testingSet*, *printResults=False*)
Function to get anomaly score of cells based on some reference set

Parameters:

trainingSet: pandas.DataFrame With cells to trail isolation forest on

testingSet: pandas.DataFrame With cells to score

printResults: boolean, Default False Whether to print results

Returns:

1d numpy.array Anomaly score(s) of tested cell(s)

Usage: DCS = DigitalCellSorter.DigitalCellSorter()

cutoff = DCS.getAnomalyScores(df_expr.iloc[:, 5:], df_expr.iloc[:, :5])

getHugoName (*gene*, *printAliases=False*)
Get gene hugo name(s).

Parameters:

gene: str 'hugo' or 'alias' name of a gene

Returns:

str Hugo name if found, otherwise input name

Usage: DCS = DigitalCellSorter.DigitalCellSorter()

DCS.getHugoName('CD138')

getExprOfGene (*gene*, *analyzeBy='cluster'*)
Get expression of a gene. Run this function only after function process()

Parameters:

cells: pandas.MultiIndex Index of cells of interest

analyzeBy: str, Default 'cluster' What level of lablels to include. Other possible options are 'label' and 'celltype'

Returns:

pandas.DataFrame With expression of the cells of interest

Usage: DCS = DigitalCellSorter.DigitalCellSorter()

DCS.process()

DCS.getExprOfGene('SDC1')

getExprOfCells (*cells*)
Get expression of a set of cells. Run this function only after function process()

Parameters:

cells: pandas.MultiIndex 2-level Index of cells of interest, must include levels 'batch' and 'cell'

Returns:

pandas.DataFrame With expression of the cells of interest

Usage: DCS = DigitalCellSorter.DigitalCellSorter()

DCS.process()

DCS.getExprOfCells(cells)

getCells (*celltype=None*, *clusterIndex=None*, *clusterName=None*)
Get cell annotations in a form of pandas.Series

Parameters:

celltype: str, Default None Cell type to extract

clusterIndex: int, Default None Cell type to extract

clusterName: str, Default None Cell type to extract

Returns:

pandas.MultiIndex Index of labelled cells

Usage: DCS = DigitalCellSorter.DigitalCellSorter()

DCS.process()

labels = DCS.getCells()

getIndexOfGoodQualityCells(*QCplotsSubDir='QC_plots'*, ***kwargs*)

Get index of sells that satisfy the QC criteria

Parameters:

count_depth_cutoff: float, Default 0.5 Fraction of median to take as count depth cutoff

number_of_genes_cutoff: float, Default 0.5 Fraction of median to take as number of genes cutoff

mitochondrial_genes_cutoff: float, Default 3.0 The cutoff is median + standard_deviation * this_parameter

Any parameters that function 'makeQualityControlHistogramPlot' can accept

Returns:

pandas.Index Index of cells

Usage: DCS = DigitalCellSorter.DigitalCellSorter()

index = DCS.getIndexOfGoodQualityCells()

getQualityControlCutoff(*se*, *cutoff*, *precut=1.0*, *mito=False*, *MakeHistogramPlot=True*, ***kwargs*)

Function to calculate QC quality cutoff

Parameters:

se: pandas.Series With data to analyze

cutoff: float Parameter for calculating the quality control cutoff

mito: boolean, Default False Whether the analysis of mitochondrial genes fraction

plotPathAndName: str, Default None Text to include in the figure title and file name

MakeHistogramPlot: boolean, Default True Whether to make a histogram plot

Any parameters that function 'makeQualityControlHistogramPlot' can accept

Returns:

float Cutoff value

Usage: DCS = DigitalCellSorter.DigitalCellSorter()

cutoff = DCS.getQualityControlCutoff(se)

getCountsDataframe(*se1*, *se2*, *tagForMissing='N/A'*)

Get a pandas.DataFrame with cross-counts (overlaps) between two pandas.Series

Parameters:

se1: pandas.Series Series with the first set of items

se2: pandas.Series Series with the second set of items

tagForMissing: str, Default 'N/A' Label to assign to non-overlapping items

Returns:

pandas.DataFrame Contains counts

Usage: DCS = DigitalCellSorter.DigitalCellSorter()

df = DCS.getCountsDataframe(se1, se2)

getNewMarkerGenes(*cluster=None*, *top=100*, *zScoreCutoff=None*, *removeUnknown=False*, ***kwargs*)

Extract new marker genes based on the cluster annotations

Parameters:

cluster: int, Default None Cluster #, if provided genes of only this culster will be returned

top: int, Default 100 Upper bound for number of new markers per cell type

zScoreCutoff: float, Default 0.3 Lower bound for a marker z-score to be significant

removeUnknown: boolean, Default False Whether to remove type "Unknown"

Any parameters that function 'makePlotOfNewMarkers' can accept

Returns: None

Usage: DCS = DigitalCellSorter.DigitalCellSorter()

DCS.extractNewMarkerGenes()

classmethod calculateV(*args*)

Calculate the voting scores (celltypes by clusters)

Parameters:

args: tuple Tuple of sub-arguments

df_M: pandas.DataFrame Marker cell type DataFrame

df_X: pandas.DataFrame Markers expression DataFrame

cluster_index: 1d numpy.array Clustering index

cutoff: float Significance cutoff, i.e. a threshold for a given marker to be significant

giveSignificant: boolean Whether to return the significance matrix along with the scores

removeLowQCscores: boolean Whether to remove low quality scores, i.e. those with less than 10% of markers that a re supporting

Returns:

pandas.DataFrame Contains voting scores per celltype per cluster

Usage: Function is used internally.

df = calculateV((df_M, df_X, cluster_index, 0.3, False, True))

annotateWith_pDCS_Scheme(*df_markers_expr*, *df_marker_cell_type*)

Produce cluster annotation results

Parameters:

df_markers_expr: pandas.DataFrame Data with marker genes by cells expression

df_marker_cell_type: pandas.DataFrame Data with marker genes by cell types

Returns: tuple

Usage: Function should be called internally only

annotateWith_ratio_Scheme(*df_markers_expr*, *df_marker_cell_type*)

Produce cluster annotation results

Parameters:

df_markers_expr: pandas.DataFrame Data with marker genes by cells expression

df_marker_cell_type: pandas.DataFrame Data with marker genes by cell types

Returns: tuple

Usage: Function should be called internally only

annotateWith_Hopfield_Scheme(*df_markers_expr*, *df_marker_cell_type*)
Produce cluster annotation results

Parameters:

df_markers_expr: pandas.DataFrame Markers expression DataFrame

df_marker_cell_type: pandas.DataFrame Marker cell type DataFrame

Returns: tuple

Usage: Function should be called internally only

recordAnnotationResults(*df_marker_cell_type*, *df_markers_expr*, *df_L*, *df_V*, *dict_expressed_markers*, *df_null_distributions=None*)
Record cell type annotation results to spreadsheets.

Parameters:

df_marker_cell_type: pandas.DataFrame Markers to cell types table

df_markers_expr: pandas.DataFrame Markers expression in each cluster

df_L: pandas.DataFrame Annotation scores along with other information

df_V: pandas.DataFrame Annotation scores along with other information

dict_expressed_markers: dictionary Dictionary of markers signigicantly expressed in each cluster

df_null_distributions: pandas.DataFrame, Default None Table with null distributions

Returns: None

Usage: This function is intended to be used internally only

propagateHopfield(*sigma=None*, *xi=None*, *T=0.2*, *tmax=200*, *fractionToUpdate=0.5*, *mode=4*, *meshSamplingRate=200*, *underlyingNetwork=None*, *typesNames=None*, *clustersNames=None*, *printInfo=False*, *recordTrajectories=True*, *id=None*, *printSwitchingFraction=False*, *path=None*, *verbose=0*)
Function is used internally to propagate Hopfield network over a set number of time steps

Parameters:

sigma: pandas.DataFrame, Default None Markers expression

xi: pandas.DataFrame, Default None Marker cell type DataFrame

T: float, Default 0.2 Noise (Temperature) parameter

tmax: int, Default 200 Number of step to iterate through

fractionToUpdate: float, Default 0.5 Fraction of nodes to randomly update at each iteration

mode: int, Default 4

Options are: 1: non-onthogonalized, non-weighted attractors 2: onthogonalized, non-weighted attractors 3: onthogonalized, weighted attractors 4: onthogonalized, weighted attractors, asymetric and diluted dynamics

meshSamplingRate: int, Default 100 Visualization parameter to control the quality of the color mesh near the attractors

underlyingNetwork: 2d numpy.array, Default None Network of underlying connections between genes

typesNames: list-like, Default None Names of cell types

clustersNames: list-like, Default None Names or identifiers of the clusters

printInfo: boolean, Default False Whether to print detailes

recordTrajectories: boolean, Default True Whether to record trajectories data to files

id: int, Default None Identifier of this function call

printSwitchingFraction: boolean, Default False Whether to print fraction of clusters that switch theie maximum overlapping attractor

path: str, Default None Path for saving trajectories data

Returns:

2d numpy.array Overlaps

Usage: result = propagateHopfield(sigma=sigma, xi=df_attrs)

classmethod convertColormap(*colormap*)

Convert colormap from the form (1.,1.,1.,1.) to 'rgba(255,255,255,1.)'

Parameters:

colormap: dictionary Colormap to convert

Returns:

dictionary Converted colomap

Usage: DCS = DigitalCellSorter.DigitalCellSorter()

colormap = DCS.convertColormap(colormap)

classmethod zScoreOfSeries(*se*)

Calculate z-score of pandas.Series and modify the Series in place

Parameters:

se: pandas.Series Series to process

Returns:

pandas.Series Processed series

Usage: DCS = DigitalCellSorter.DigitalCellSorter()

se = DCS.zScoreOfSeries(se)

classmethod KeyInFile(*key*, *file*)

Check is a key exists in a HDF file.

Parameters:

key: str Key name to check

file: str HDF file name to check

Returns:

boolean True if the key is found False otherwise

Usage: DCS = DigitalCellSorter.DigitalCellSorter()

DCS.KeyInFile('df_expr', 'data/file.h5')

getSubnetworkOfPCN(*subnetworkGenes*, *min_shared_first_targets=30*)
Extract subnetwork of PCN network

Parameters:

subnetworkGenes: list-like Set of genes that the subnetwork should contain

min_shared_first_targets: int, Default 30 Number of minimum first shared targets to connect two nodes

Returns:

pandas.DataFrame Adjacency matrix

Usage: DCS = DigitalCellSorter.DigitalCellSorter()

df_subnetwork = DCS.getSubnetworkOfPCN(genes)

alignSeries(*se1*, *se2*, *tagForMissing*)
Align two pandas.Series

Parameters:

se1: pandas.Series Series with the first set of items

se2: pandas.Series Series with the second set of items

tagForMissing: str, Default 'Missing' Label to assign to non-overlapping items

Returns:

pandas.DataFrame Contains two aligned pandas.Series

Usage: DCS = DigitalCellSorter.DigitalCellSorter()

df = DCS.alignSeries(pd.Index(['A', 'B', 'C', 'D']).to_series(), pd.Index(['B', 'C', 'D', 'E', 'F']).to_series())

createReverseDictionary(*inputDictionary*)
Efficient way to create a reverse dictionary from a dictionary. Utilizes Pandas.Dataframe.groupby and Numpy arrays indexing.

Parameters:

inputDictionary: dictionary Dictionary to reverse

Returns:

dictionary Reversed dictionary

Usage: DCS = DigitalCellSorter.DigitalCellSorter()

revDict = DCS.createReverseDictionary(Dict)

readMarkerFile(*mergeFunction='mean'*, *mergeCutoff=0.25*)
Read markers file, prepare markers

Parameters:

mergeCutoff: str, Default 'mean'

Function used for grouping of the cell sub-types. Options are: 'mean': average of the values 'max': maxium of the values, effectively a logiacal OR function

mergeCutoff: float, Default 0.25 Values below cutoff are set to zero. This option is used if mergeCutoff is 'mean'

Returns:

pandas.DataFrame Celltype/markers matrix

Usage: DCS = DigitalCellSorter.DigitalCellSorter()

df_marker_cell_type = DCS.readMarkerFile()

mergeIndexDuplicates (*df_expr*, *method='average'*, *printDuplicates=False*, *verbose=1*)
Merge index duplicates

Parameters:

df_expr: pandas.DataFrame Gene expression table

method: str, Default None

How to deal with index duplicates. Option are: 'average': average values of duplicates

'first': keep only first of duplicates, discard rest

Returns:

pandas.DataFrame Gene expression table

Usage: DCS = DigitalCellSorter.DigitalCellSorter()

df_expr = DCS.mergeIndexDuplicates(df_expr)

recordExpressionData ()
Record expression data from the internal HDF storage.

Parameters: None

Returns: None

Usage: DCS = DigitalCellSorter.DigitalCellSorter()

DCS.recordExpressionData()

loadAnnotatedLabels (*detailed=False*, *includeLowQC=True*, *infoType='label'*)
Load cell annotations resulted from function 'annotate'

Parameters:

detailed: boolean, Default False Whether to give cluster- or celltype- resolution data

includeLowQC: boolean, Default False Whether to include low quality cells in the output

Returns: pandas.Series

Usage: DCS = DigitalCellSorter.DigitalCellSorter()

DCS.loadAnnotatedLabels()

loadExpressionData ()
Load processed expression data from the internal HDF storage.

Parameters: None

Returns: None

Usage: DCS = DigitalCellSorter.DigitalCellSorter()

DCS.loadExpressionData()

prepareMarkers(*expressedGenes=None*, *createColormapForCelltypes=True*)
Get dictionary of markers for each cell types.

Parameters:

expressedGenes: pandas.Index, Default None If not None then the marker DataFrame will be intersected with this index, i.e. all non-expressed genes will be filtered from the marker file

createColormapForCelltypes: boolean, Default True Create (or update) a colormap for cell types based on a marker-celltype matrix. This will make coloring of cell clusters consistent across all plots.

Usage: DCS = DigitalCellSorter.DigitalCellSorter()

DCS.prepareMarkers()

calculateQCmeasures()
Calculate Quality Control (QC) measures

Parameters: None

Returns: None

Usage: DCS = DigitalCellSorter.DigitalCellSorter()

DCS.calculateQCmeasures()

qualityControl(***kwargs*)
Remove low quality cells

Parameters: None

Returns: Any parameters that function 'getIndexOfGoodQualityCells' can accept

Usage: DCS = DigitalCellSorter.DigitalCellSorter()

DCS.qualityControl()

batchEffectCorrection(*method='COMBAT'*)
Batch effect correction.

Parameters:

method: str, Default 'COMBAT' Stein, C.K., Qu, P., Epstein, J. et al. Removing batch effects from purified plasma cell gene expression microarrays with modified ComBat. BMC Bioinformatics 16, 63 (2015)

Returns: None

Usage: DCS = DigitalCellSorter.DigitalCellSorter()

DCS.batchEffectCorrection()

CHAPTER FIVE

VISUALIZATION FUNCTIONS API

The package contains a set of visualization functions that are used at the stage of post-processing.

Note: These functions are not intended to be accessed directly by a user. See **User Functions** for the list of visualization tools that utilize visualization functions detailed here.

Submodule VisualizationFunctions

5.1 Cell type markers pie plot

This visualization function can be launched from *class DigitalCellSorter* at the stage of pre- and post-processing.

From submodule *VisualizationFunctions*:

class VisualizationFunctions(*dataName='dataName'*, *saveDir=''*, *matplotlibMode='Agg'*, *safePlotting=True*, *verbose=1*)

Class of visualization functions for DigitalCellSorter

makeCellMarkersPiePlot(**args*, ***kwargs*)

Example output:

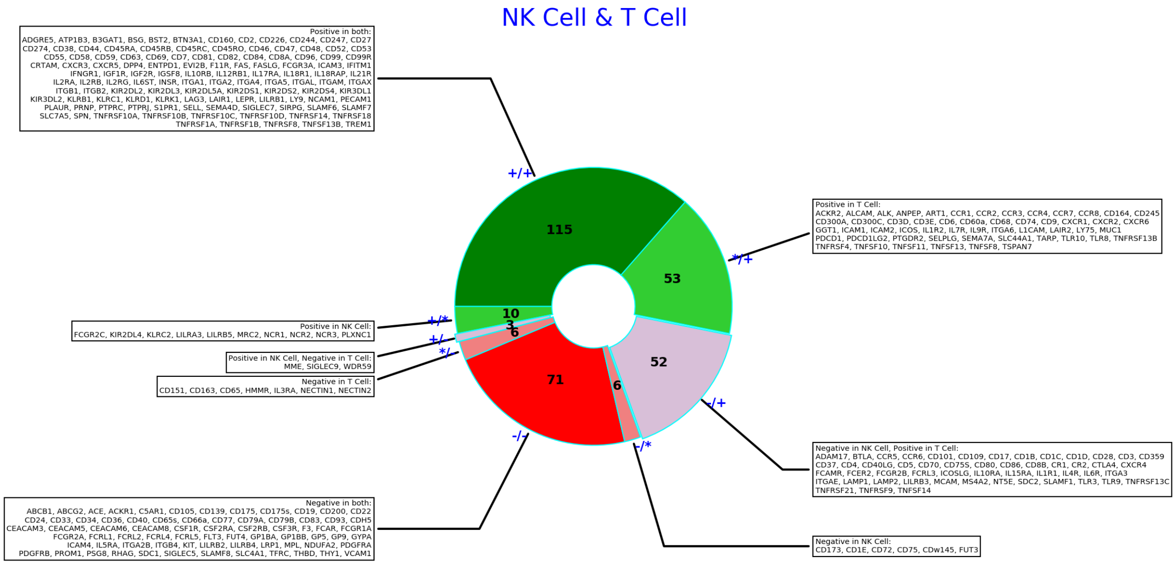

5.2 Projection plot

This visualization function can be launched from *class DigitalCellSorter* at the stage of post-processing.

From submodule *VisualizationFunctions*:

class VisualizationFunctions(*dataName='dataName'*, *saveDir="*, *matplotlibMode='Agg'*, *safePlotting=True*, *verbose=1*)

Class of visualization functions for DigitalCellSorter

makeProjectionPlot(**args*, ***kwargs*)

Example output:

5.3 Marker subplots

This visualization function can be launched from *class DigitalCellSorter* at the stage of post-processing.

From submodule *VisualizationFunctions*:

class VisualizationFunctions(*dataName='dataName'*, *saveDir="*, *matplotlibMode='Agg'*, *safePlotting=True*, *verbose=1*)

Class of visualization functions for DigitalCellSorter

internalMakeMarkerSubplots(**args*, ***kwargs*)

Example output:

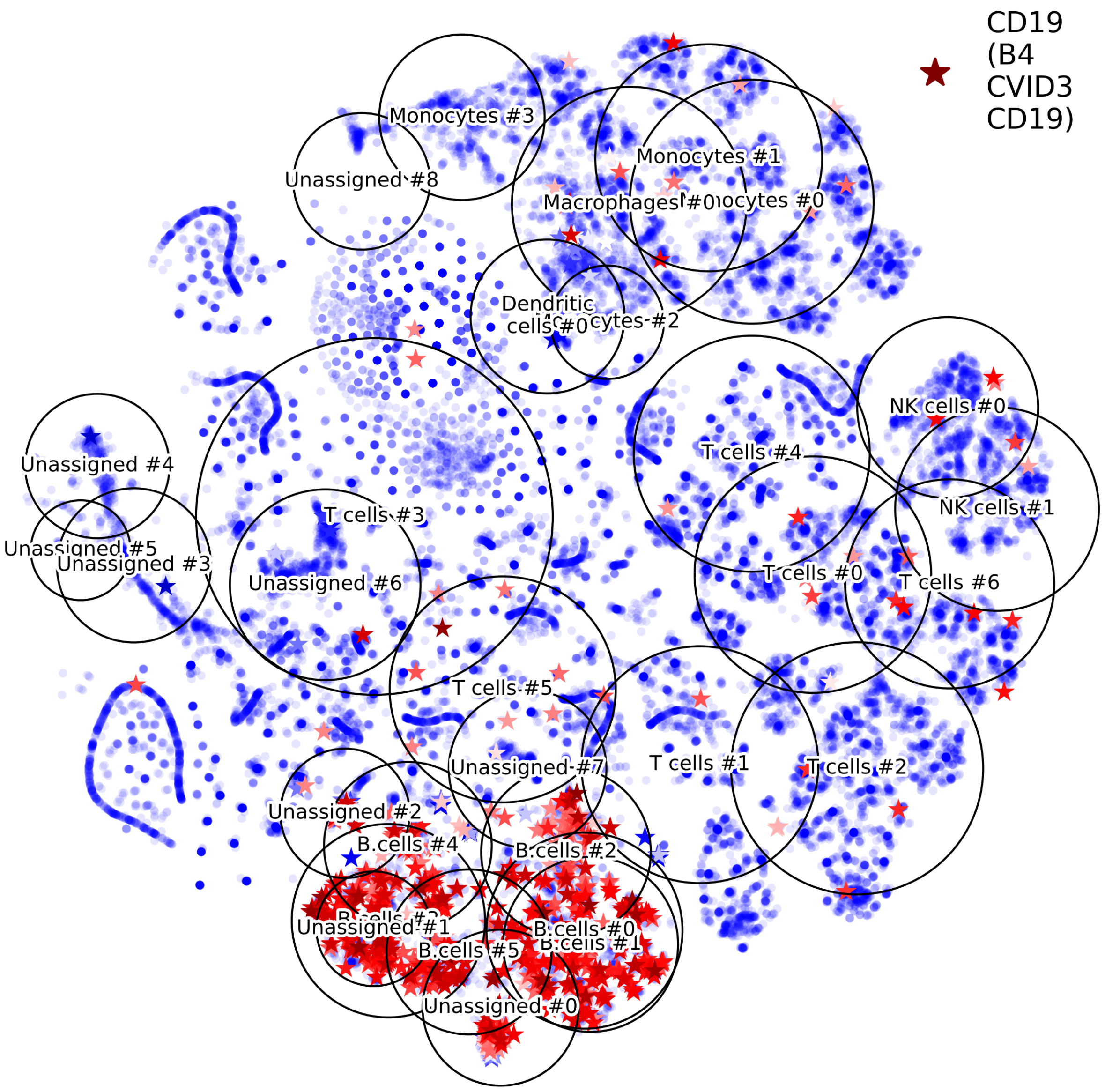

5.4 Quality control histogram plot

This visualization function can be launched from *class DigitalCellSorter* at the stage of post-processing.

From submodule *VisualizationFunctions*:

class VisualizationFunctions(*dataName='dataName'*, *saveDir=''*, *matplotlibMode='Agg'*, *safePlotting=True*, *verbose=1*)

Class of visualization functions for DigitalCellSorter

makeQualityControlHistogramPlot(**args*, ***kwargs*)

Example output:

5.5 Histogram null distribution plot

This visualization function can be launched from *class DigitalCellSorter* at the stage of post-processing.

From submodule *VisualizationFunctions*:

class VisualizationFunctions(*dataName='dataName'*, *saveDir="*, *matplotlibMode='Agg'*, *safePlotting=True*, *verbose=1*)

Class of visualization functions for DigitalCellSorter

makeHistogramNullDistributionPlot(**args*, ***kwargs*)

Example output:

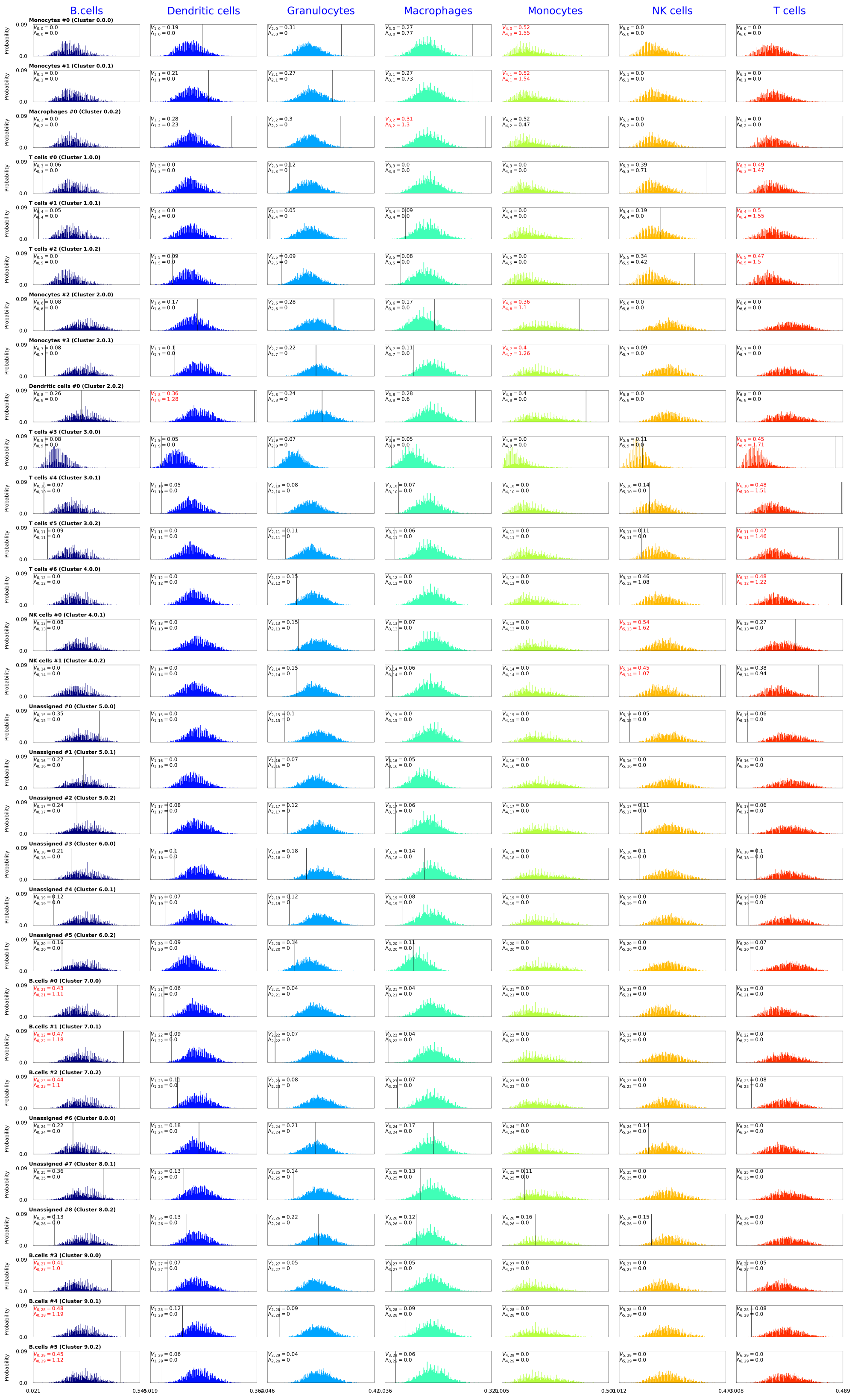

5.6 Sankey diagram

This visualization function can be launched from *class DigitalCellSorter* at the stage of post-processing.

From submodule *VisualizationFunctions*:

class VisualizationFunctions(*dataName='dataName'*, *saveDir="*, *matplotlibMode='Agg'*, *safePlotting=True*, *verbose=1*)

Class of visualization functions for DigitalCellSorter

makeSankeyDiagram(**args*, ***kwargs*)

Example output:

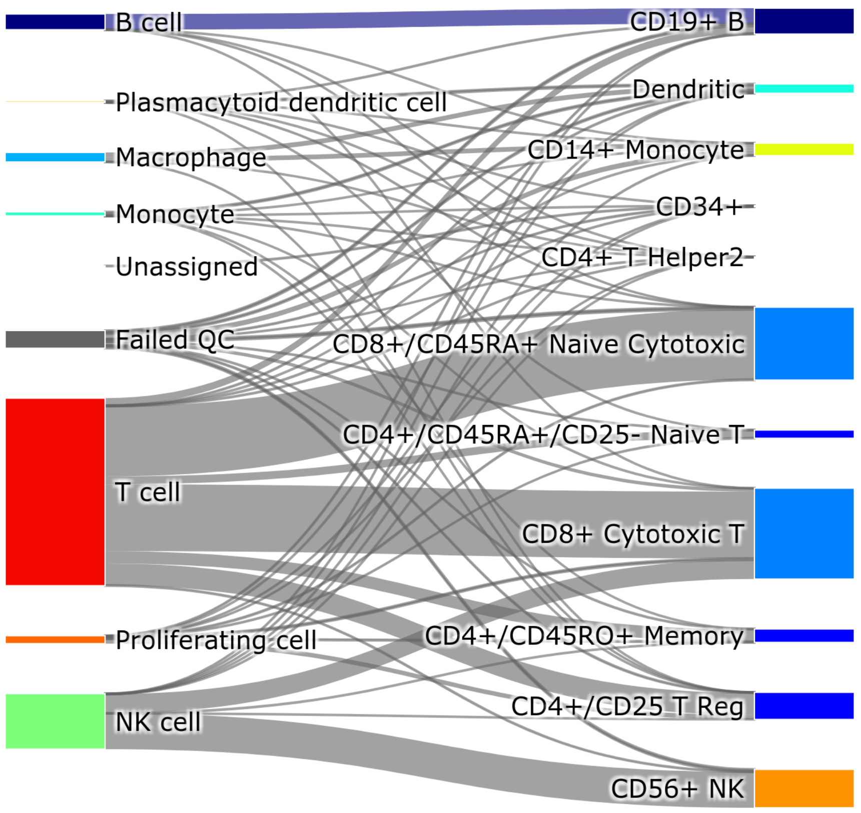

5.7 Stacked bar plot

This visualization function can be launched from *class DigitalCellSorter* at the stage of post-processing.

From submodule *VisualizationFunctions*:

class VisualizationFunctions(*dataName='dataName'*, *saveDir=''*, *matplotlibMode='Agg'*, *safePlotting=True*, *verbose=1*)
Class of visualization functions for DigitalCellSorter

makeStackedBarplot(**args*, ***kwargs*)

Example output:

5.8 Annotation Results Matrix plot

This visualization function can be launched from *class DigitalCellSorter* at the stage of post-processing.

From submodule *VisualizationFunctions*:

class VisualizationFunctions(*dataName='dataName'*, *saveDir=''*, *matplotlibMode='Agg'*, *safePlotting=True*, *verbose=1*)
Class of visualization functions for DigitalCellSorter

makeAnnotationResultsMatrixPlot(**args*, ***kwargs*)

Example output:

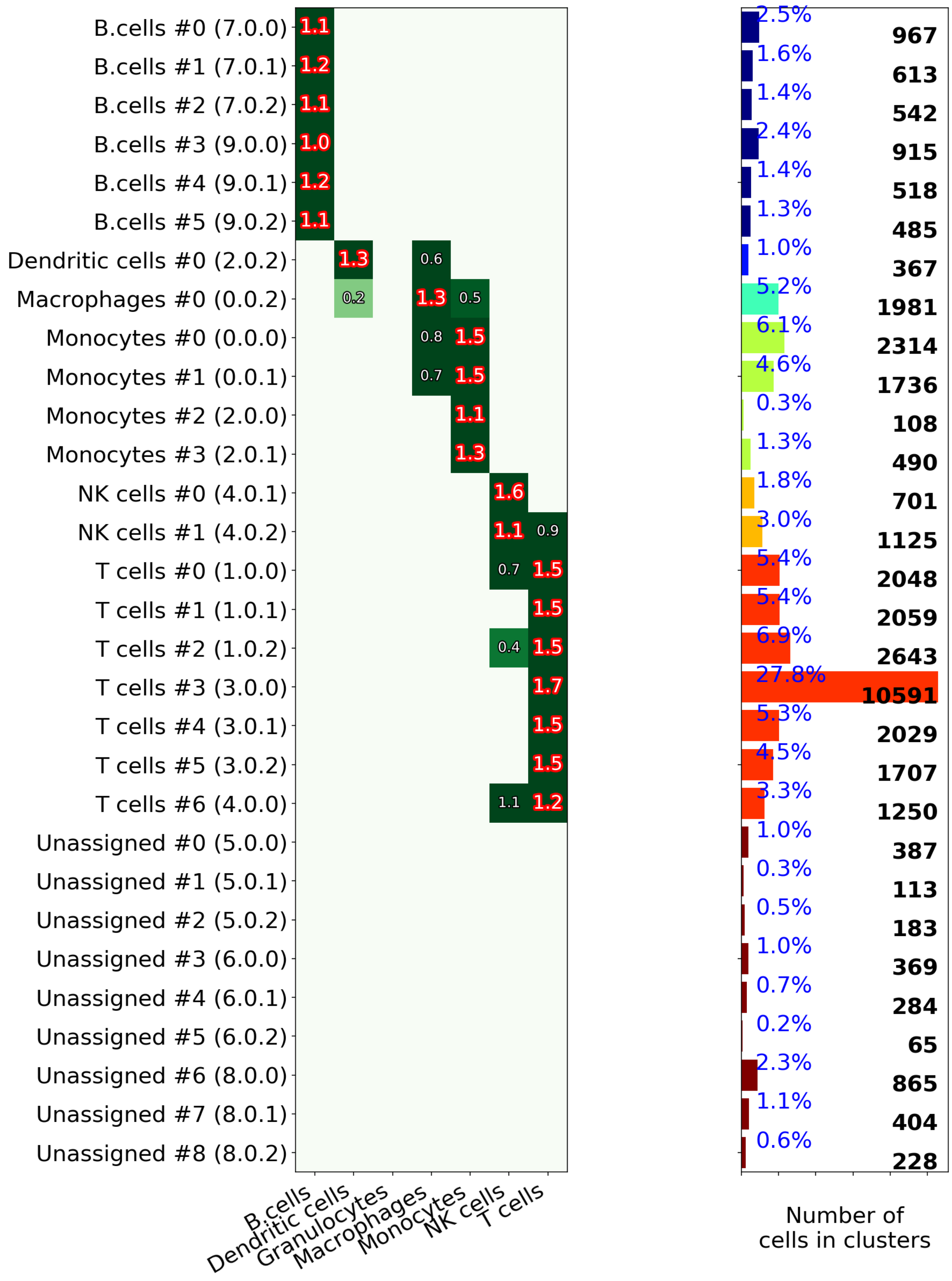
B.cells #0 (7.0.0)
B.cells #1 (7.0.1)
B.cells #2 (7.0.2)
B.cells #3 (9.0.0)
B.cells #4 (9.0.1)
B.cells #5 (9.0.2)
Dendritic cells #0 (2.0.2)
Macrophages #0 (0.0.2)
Monocytes #0 (0.0.0)
Monocytes #1 (0.0.1)
Monocytes #2 (2.0.0)
Monocytes #3 (2.0.1)
NK cells #0 (4.0.1)
NK cells #1 (4.0.2)
T cells #0 (1.0.0)
T cells #1 (1.0.1)
T cells #2 (1.0.2)
T cells #3 (3.0.0)
T cells #4 (3.0.1)
T cells #5 (3.0.2)
T cells #6 (4.0.0)
Unassigned #0 (5.0.0)
Unassigned #1 (5.0.1)
Unassigned #2 (5.0.2)
Unassigned #3 (6.0.0)
Unassigned #4 (6.0.1)
Unassigned #5 (6.0.2)
Unassigned #6 (8.0.0)
Unassigned #7 (8.0.1)
Unassigned #8 (8.0.2)
B.cells
Dendritic cells
Granulocytes
Macrophages
Monocytes
NK cells
T cells
Number of cells in clusters
2.5% 967
1.6% 613
1.4% 542
2.4% 915
1.4% 518
1.3% 485
1.0% 367
5.2% 1981
6.1% 2314
4.6% 1736
0.3% 108
1.3% 490
1.8% 701
3.0% 1125
5.4% 2048
5.4% 2059
6.9% 2643
27.8% 10591
5.3% 2029
4.5% 1707
3.3% 1250
1.0% 387
0.3% 113
0.5% 183
1.0% 369
0.7% 284
0.2% 65
2.3% 865
1.1% 404
0.6% 228

5.9 Marker expression plot

This visualization function can be launched from *class DigitalCellSorter* at the stage of post-processing.

From submodule *VisualizationFunctions*:

class VisualizationFunctions(*dataName='dataName'*, *saveDir=''*, *matplotlibMode='Agg'*, *safePlotting=True*, *verbose=1*)

Class of visualization functions for DigitalCellSorter

makeMarkerExpressionPlot(**args*, ***kwargs*)

Example output:

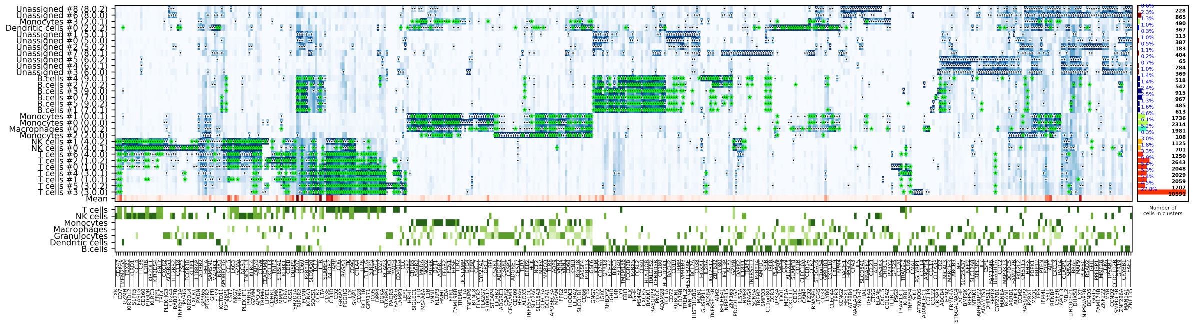

5.10 t-test plot

This visualization function can be launched from *class DigitalCellSorter* at the stage of post-processing.

From submodule *VisualizationFunctions*:

class VisualizationFunctions(*dataName='dataName'*, *saveDir=''*, *matplotlibMode='Agg'*, *safePlotting=True*, *verbose=1*)

Class of visualization functions for DigitalCellSorter

makeTtestPlot(**args*, ***kwargs*)

Example output:

CD4
(CD4
CD4mut)

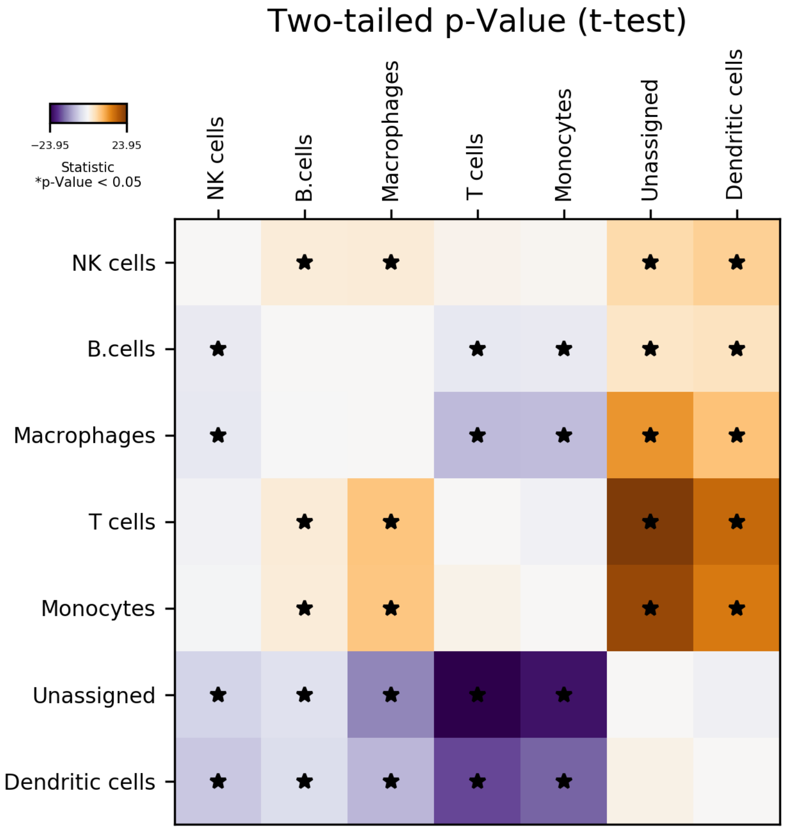

5.11 Plot of new markers

This visualization function can be launched from *class DigitalCellSorter* at the stage of post-processing.

From submodule *VisualizationFunctions*:

class VisualizationFunctions(*dataName='dataName'*, *saveDir=''*, *matplotlibMode='Agg'*, *safePlotting=True*, *verbose=1*)

Class of visualization functions for DigitalCellSorter

makePlotOfNewMarkers(**args*, ***kwargs*)

Example output:

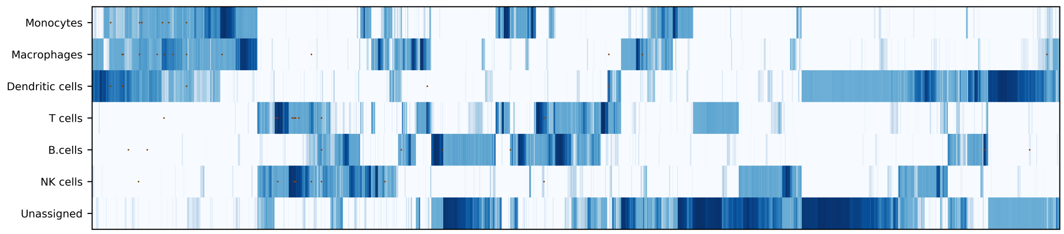

CHAPTER SIX

GENERIC FUNCTIONS

A set of generic tools grouped here are used by the Class of DigitalCellSorter.

Submodule GenericFunctions

Ggeneral functions for conveniece of use

Functions:

extractFromZipOfGz(filepath[, . . .])	
getElapsedTime(start)	Print total elapsed time (in minutes) elapsed from the reference point
getStartTime()	Get time (in seconds) elapsed from the epoch
read(fileName[, compressed, jsonFormat])	Unpickle object from a (binary) file
timeMark()	Print total time elapsed from the beggining of the process from which the function is called
write(data, fileName[, compressed, jsonFormat])	Pickle object into a (binary) file

write(*data*, *fileName*, *compressed=False*, *jsonFormat=False*)

Pickle object into a (binary) file

Parameters: data: any Pyhton object, e.g. list, dictionary, file, method, variable, etc. fileName: path and name of the file to store binary data in

Returns: None

Usage: data = [['A', 'B', 'C'], pd.DataFrame()] write(data, os.path.join('some dir 1', 'some dir 2', 'File with my data'))

read(*fileName*, *compressed=False*, *jsonFormat=False*)

Unpickle object from a (binary) file

Parameters: fileName: path and name of the file with binary data stored in

Returns: Data stored in the provided file

Usage: read(os.path.join('some dir 1', 'some dir 2', 'File with my data'))

timeMark()

Print total time elapsed from the beggining of the process from which the function is called

Parameters: None

Returns: None

Usage: timeMark()

getStartTime()

Get time (in seconds) elapsed from the epoch

Parameters: None

Returns: Time (in seconds)

Usage: start = getStartTime()

getElapsedTime (*start*)

Print total elapsed time (in minutes) elapsed from the reference point

Parameters:

start: float or int Reference time (in seconds)

Returns: None

Usage: getElapsedTime(start)

extractFromZipOfGz (*filepath*, *removeDownloadedZipFile=False*)

CHAPTER SEVEN

DEPENDENCIES

This graph was generated with **Python** module dependency visualization tool `pydeps`, see GitHub, by running the following (after installation of the necessary components):

```
pydeps DigitalCellSorter --reverse --max-bacon=2 --cluster --max-cluster-size=6 --min-
↪cluster-size=2 -T=png -o=docs/DigitalCellSorter_pydeps_current.png
```

CHAPTER

EIGHT

DATA PREPARATION

8.1 Output from kallisto-bustools (kp-python)

In this example we use raw sequencing data stored in `.fastq` format, from 1000 PBMC, the data can be accessed at https://support.10xgenomics.com/single-cell-gene-expression/datasets/3.0.0/pbmc_1k_v3

Note: This is by no means a tutorial for processing scRNA-seq data. We only demonstrate the workflow of connecting upstream analysis software and DCS.

Download the data and unpack the `.tar` file (~5.17 GB):

```
wget https://cf.10xgenomics.com/samples/cell-exp/3.0.0/pbmc_1k_v3/pbmc_1k_v3_fastqs.
↪tar
tar -xvf pbmc_1k_v3_fastqs.tar
```

To process sequencing data one could use `kallisto bus` tool to generate BUS file following by `bustools count` to generate count matrices from a BUS file. However, we prefer to use `kb-python`, a package that wraps the `kallisto` and `bustools` single-cell RNA-seq workflow (Bray, N. L., Pimentel, H., Melsted, P., & Pachter, L. (2016). Near-optimal probabilistic RNA-seq quantification. Nature biotechnology, 34(5), 525) `kb-python` can be installed with `pip`.

```
kb count -i kallisto_index/homo_sapiens/transcriptome.idx \
         -g kallisto_index/homo_sapiens/transcripts_to_genes.txt \
         -x 10xv3 \
         --filter \
         -t 4 \
         pbmc_1k_v3_fastqs/pbmc_1k_v3_S1_L001_R1_001.fastq.gz \
         pbmc_1k_v3_fastqs/pbmc_1k_v3_S1_L001_R2_001.fastq.gz \
         pbmc_1k_v3_fastqs/pbmc_1k_v3_S1_L002_R1_001.fastq.gz \
         pbmc_1k_v3_fastqs/pbmc_1k_v3_S1_L002_R2_001.fastq.gz
```

Output from kb count command above

[2020-11-20 14:32:51,136] INFO Using index kallisto_index/homo_sapiens/transcriptome.idx to generate BUS file to . from

[2020-11-20 14:32:51,136] INFO pbmc_1k_v3_fastqs/pbmc_1k_v3_S1_L001_R1_001.fastq.gz

[2020-11-20 14:32:51,136] INFO pbmc_1k_v3_fastqs/pbmc_1k_v3_S1_L001_R2_001.fastq.gz

[2020-11-20 14:32:51,136] INFO pbmc_1k_v3_fastqs/pbmc_1k_v3_S1_L002_R1_001.fastq.gz

[2020-11-20 14:32:51,136] INFO pbmc_1k_v3_fastqs/pbmc_1k_v3_S1_L002_R2_001.fastq.gz

[2020-11-20 14:36:33,477] INFO Sorting BUS file ./output.bus to ./tmp/output.s.bus

[2020-11-20 14:36:57,118] INFO Whitelist not provided

[2020-11-20 14:36:57,118] INFO Copying pre-packaged 10XV3 whitelist to .

[2020-11-20 14:36:57,675] INFO Inspecting BUS file ./tmp/output.s.bus

[2020-11-20 14:37:07,641] INFO Correcting BUS records in ./tmp/output.s.bus to ./tmp/output.s.c.bus with whitelist ./10xv3_whitelist.txt

[2020-11-20 14:37:29,264] INFO Sorting BUS file ./tmp/output.s.c.bus to ./output.unfiltered.bus

[2020-11-20 14:37:47,478] INFO Generating count matrix ./counts_unfiltered/cells_x_genes from BUS file ./output.unfiltered.bus

[2020-11-20 14:37:59,662] INFO Filtering with bustools

[2020-11-20 14:37:59,662] INFO Generating whitelist ./filter_barcodes.txt from BUS file ./output.unfiltered.bus

[2020-11-20 14:37:59,790] INFO Correcting BUS records in ./output.unfiltered.bus to ./tmp/output.unfiltered.c.bus with whitelist ./filter_barcodes.txt

[2020-11-20 14:38:14,344] INFO Sorting BUS file ./tmp/output.unfiltered.c.bus to ./output.filtered.bus

[2020-11-20 14:38:30,918] INFO Generating count matrix ./counts_filtered/cells_x_genes from BUS file ./output.filtered.bus

The output directory that we are interested in is `counts_filtered/`. Rename it:

```
mv counts_filtered/ kb_1k_PBMC_output/
```

This will generate counts data in the directory `kb_1k_PBMC_output/`.

8.2 Output from CellRanger

Here we use CellRanger-processed data stored in `.mtx` format, from 1000 PBMC, the data can be accessed at https://support.10xgenomics.com/single-cell-gene-expression/datasets/3.0.0/pbmc_1k_v3

Download the data and unpack the `.tar.gz` file (~9 MB):

```
wget https://cf.10xgenomics.com/samples/cell-exp/3.0.0/pbmc_1k_v3/pbmc_1k_v3_filtered_
↪feature_bc_matrix.tar.gz
tar -xzf pbmc_1k_v3_filtered_feature_bc_matrix.tar.gz && mv filtered_feature_bc_
↪matrix/ cellranger_1k_PBMC_output/
```

These two commands will prepare the processed counts data in the directory `cellranger_1k_PBMC_output/`.

8.3 Import from kallisto-bustools (kp-python)

```
import DigitalCellSorter
from DigitalCellSorter.core import readMTXdata

# Read the MTX data
df = readMTXdata(dataDir='kb_1k_PBMC_output/', origin='kb-python')

# (Optional) Convert gene names to HUGO
DCS = DigitalCellSorter.DigitalCellSorter()
DCS.prepare(df)
DCS.convert('ensembl', 'hugo')

# Check the DCS data
print(DCS.df_expr)
```

8.4 Import from CellRanger

```
import DigitalCellSorter
from DigitalCellSorter.core import readMTXdata

# Read the MTX data
df = readMTXdata(dataDir='cellranger_1k_PBMC_output/', origin='cellranger')

# (Optional) Convert gene names to HUGO
DCS = DigitalCellSorter.DigitalCellSorter()
DCS.prepare(df)
DCS.convert('ensembl', 'hugo')

# Check the DCS data
print(DCS.df_expr)
```

8.5 Function readMTXdata

Function to read data in MTX format (see usage examples above).

readMTXdata (*dataDir*, *origin*, *fileMatrix=None*, *fileBarcodes=None*, *fileGenes=None*, *headerRows=None*, *sampleName=None*, *stripGeneVersions=True*, *saveData=True*, *dropGeneDuplicates=True*, *dropCellDuplicates=True*)

Read MTX format into pandas DataFrame compatible with DCS input format

Parameters

dataDir: str Path to gene expression counts data

origin: str

Name of the software where the data was generated. Supported options are: 'kb-python' for kallisto-bustools 'cellranger' for cellRanger

fileMatrix: str, Default None Name of the matrix file

fileBarcodes: str, Default None Name of the cell barcodes file

fileGenes: str, Default None Name of the genes file

headerRows: list, Default None List of rows in matrix file to skip

sampleName: str, Default None Name of the data sample to include in the batch level

stripGeneVersions: boolean, Default True Remove ensembl gene version. E.g. "ENSG00000236246.1" –> "ENSG00000236246"

saveData: boolean, Default True Whether to save data in hdf format. If True then the data is saved to a compressed hdf at the same location as matrix data

dropGeneDuplicates: boolean, Default True Whether to remove gene duplicates (keep first)

dropGeneDuplicates: boolean, Default True Whether to remove barcode duplicates (keep first)

Returns:

pandas.DataFrame Table that has genes in rows and cells in columns

Usage: df = readMTX(dataDir='filtered_feature_bc_matrix/', origin='cellranger') #df = readMTX(dataDir='counts_filtered/', origin='kb-python')

DCS = DigitalCellSorter.DigitalCellSorter() DCS.prepare(df) DCS.convert('ensembl', 'hugo') print(DCS.df_expr)

8.6 Human Cell Atlas tools

Set of generic tools for retrieving, loading, and preparation of Human Cell Atlas (HCA) datasets is contained in this module.

Example:

```
import os
import DigitalCellSorter.ReadPrepareDataHCA as prep

# Example URL of a relatvely small dataset of scRNA-seq of human pancreas
url = "https://data.humancellatlas.org/project-assets/project-matrices/cddab57b-6868-
↪4be4-806f-395ed9dd635a.homo_sapiens.mtx.zip"

# Path of directories where the data will be placed
extractPath = os.path.join(os.path.join(os.path.dirname(__file__), ''), 'data', os.
↪path.splitext(os.path.basename(url))[0])

# Download data and unpack it to a specified directory
prep.getHCAdataByURL(url, extractPath)

# Record *.h5 files of individual donor IDs
IDs = prep.recordFilesOfIndividualDonors(extractPath, organName='islet of Langerhans')

# Load ready-to-use dataset of the first donor ID
df = prep.getDataframeByDonorID(extractPath, IDs[0])

# Print the shape of just loaded dataset
print(df.shape)
```

Submodule ReadPrepareDataHCA

Functions:

PrepareDataOnePatient_PREVIEW_DATASET(. . . [, . . .])	Prepare data from Human Cell Atlas (HCA) preview dataset h5 data file.
extractFromZipOfGz(filepath[, . . .])	
getDataframeByDonorID(extractPath, donorID)	Get pandas.DataFrame by Donor ID
getHCAdataByURL(url, extractPath[, extractData])	Download and extract data from Human Cell Atlas Portal
prepareDemo5kData(dir)	
read(fileName[, compressed, jsonFormat])	Unpickle object from a (binary) file
recordFilesOfIndividualDonors(extractPath[, . . .])	Record h5 files of HCA individual donors in a dataset
write(data, fileName[, compressed, jsonFormat])	Pickle object into a (binary) file

getHCAdataByURL(*url*, *extractPath*, *extractData=True*)

Download and extract data from Human Cell Atlas Portal

Parameters:

url: str URL of the data of interest

extractPath: str Path where to save and extract data to

extractData: boolean, Default True Whether to extract downloaded data

Returns: None

Usage: getHCAdataByURL(url, extractPath)

recordFilesOfIndividualDonors(*extractPath*, *organName=None*, *donorIDcolumn='donor_organism.provenance.document_id'*, *organColumn='derived_organ_parts_label'*, *useHogoGeneNames=True*)

Record h5 files of HCA individual donors in a dataset

Parameters:

extractPath: str Path of directories where HCA matrix files were downloaded and extracted. See function getHCAdataByURL() for detail.

organName: str, Default None Name of the organ name. E.g. 'pancreas', 'bone marrow', etc.

donorIDcolumn: str, Default donor_organism.provenance.document_id Column with unique IDs of donors in the file. Another option is 'specimen_from_organism.provenance.document_id' IDs at samples level is needed.

organColumn: str, Default 'derived_organ_parts_label' 'derived_organ_label'

'derived_organ_parts_label' This option is ignored when organName parameter is None.

useHogoGeneNames: boolean, Default True Whether to use HUGO gene names.

Returns:

list List of donor IDs

Usage: recordFilesOfIndividualDonors(extractPath, organName='retina')

getDataframeByDonorID(*extractPath*, *donorID*)

Get pandas.DataFrame by Donor ID

Parameters:

extractPath: str Path of directories where HCA matrix files were downloaded and extracted. See function getHCAdataByURL() for detail.

donorID: str Donor ID.

Returns:

pandas.DataFrame Matrix corresponding to the Donor ID

Usage: getDataframeByDonorID(extractPath, donorID)

PrepareDataOnePatient_PREVIEW_DATASET (*filename*, *patient*, *saveFolderName*, *useAllData=True*, *cellsLimitToUse=1000*, *randomlySample=True*, *randomSeed=0*)

Prepare data from Human Cell Atlas (HCA) preview dataset h5 data file. The user can download the file ica_bone_marrow_h5.h5 from https://preview.data.humancellatlas.org/ (Raw Counts Matrix - Bone Marrow) and place in folder data. The file is ~485Mb and contains all 378000 cells from 8 bone marrow donors (BM1-BM8). Note: this data file is no longer available at HCA data server, however, some users may have a copy of it and need to extract data from it.

Parameters:

filename: str Path and name of the file to store binary data in

patient: str Identifier of the patient: 'BM1', 'BM2', 'BM3', 'BM4', 'BM5', 'BM6', 'BM7' or 'BM8'

saveFolderName: str Path where to save prepared data file

useAllData: boolean, Default True Whether to use all data or a subset

cellsLimitToUse: int, Default 1000 Number of cells to use if useAllData=False

randomlySample: boolean, Default True Whether to sample cell randomly of pick top number

randomSeed: int, Default 0 Random seed

Returns: None

Usage: PrepareDataOnePatient(os.path.join('data', 'ica_bone_marrow_h5.h5'), 'BM1', os.path.join('data', ''), useAllData=False, cellsLimitToUse=5000)

prepareDemo5kData (*dir*)

CHAPTER NINE

INPUT DATA FORMAT

Gene Expression Data Format

The input gene expression data is expected in one of the following formats:

1. Spreadsheet of comma-separated values `csv` containing condensed matrix in a form `('cell', 'gene', 'expr')`. If there are batches in the data the matrix has to be of the form `('batch', 'cell', 'gene', 'expr')`. Columns order can be arbitrary.

cell	gene	expr
C1	G1	3
C1	G2	2
C1	G3	1
C2	G1	1
C2	G4	5
…	…	…

or:

batch	cell	gene	expr
batch0	C1	G1	3
batch0	C1	G2	2
batch0	C1	G3	1
batch1	C2	G1	1
batch1	C2	G4	5
…	…	…	…

2. Spreadsheet of comma-separated values `csv` where rows are genes, columns are cells with gene expression counts. If there are batches in the data the spreadsheet the first row should be `'batch'` and the second `'cell'`.

cell	C1	C2	C3	C4
G1		3	1	7
G2	2	2		2
G3	3	1		5
G4	10		5	4
…	…	…	…	…

or:

batch	batch0	batch0	batch1	batch1
cell	C1	C2	C3	C4
G1		3	1	7
G2	2	2		2
G3	3	1		5
G4	10		5	4
…	…	…	…	…

3. `Pandas DataFrame` where `axis 0` is genes and `axis 1` are cells. If the are batched in the data then the index of `axis 1` should have two levels, e.g. `('batch', 'cell')`, with the first level indicating patient, batch or expreriment where that cell was sequenced, and the second level containing cell barcodes for identification.

```
df = pd.DataFrame(data=[[2,np.nan],[3,8],[3,5],[np.nan,1]],
                  index=['G1','G2','G3','G4'],
                  columns=pd.MultiIndex.from_arrays([['batch0','batch1'],['C1','C2']],
↪ names=['batch', 'cell']))
```

4. `Pandas Series` where index should have two levels, e.g. `('cell', 'gene')`. If there are batched in the data the first level should be indicating patient, batch or expreriment where that cell was sequenced, the second level cell barcodes for identification and the third level gene names.

```
se = pd.Series(data=[1,8,3,5,5],
               index=pd.MultiIndex.from_arrays([['batch0','batch0','batch1','batch1',
↪'batch1'],
                                                 ['C1','C1','C1','C2','C2'],
                                                 ['G1','G2','G3','G1','G4']], names=[
↪'batch', 'cell', 'gene']))
```

Any of the data types outlined above need to be prepared/validated with a function `prepare()`.

CHAPTER

TEN

DEMO

```python
import sys
sys.path.append("..")

import os
import DigitalCellSorter
import DigitalCellSorter.ReadPrepareDataHCA as prep

if __name__ == '__main__':

    here = os.path.dirname(__file__)

    url = "https://data.humancellatlas.org/project-assets/project-matrices/cc95ff89-
↪2e68-4a08-a234-480eca21ce79.homo_sapiens.mtx.zip"
    extractPath = os.path.join(here, 'data', os.path.splitext(os.path.
↪basename(url))[0])

    # Download and unpack data
    prep.getHCAdataByURL(url, extractPath)

    # Call function recordFilesOfIndividualDonors to load the data from HCA Data␣
↪Portal
    id = prep.recordFilesOfIndividualDonors(extractPath, organName='bone marrow')[0]

    # Load gene expression data from h5 file
    df_expr = prep.getDataframeByDonorID(extractPath, id)
    df_expr.columns.names = ['batch', 'cell']

    # Create an instance of class DigitalCellSorter.
    # Here we use Default parameter values for most of the parameters
    DCS = DigitalCellSorter.DigitalCellSorter(dataName='BM1',
                                              saveDir=os.path.join(here, 'output',
↪'BM1', ''),
                                              geneListFileName='CIBERSORT_LM22_7')

    # Validate the expression data, so that it has correct form
    DCS.prepare(df_expr)

    # Delete df_expr as now DCS contains the master copy of it
    del df_expr

    # Process the expression data, i.e. quality control, dimensionality reduction,␣
↪clustering
    DCS.process()
```

(continues on next page)

(continued from previous page)

```
    # Load marker genes and annotate cells
    DCS.annotate()

    # Make plots of annotated data
    DCS.visualize()

    # Make CD19 gene expression plot
    for name in DCS.getHugoName('CD19'):
        DCS.makeIndividualGeneExpressionPlot(name)

    # Make CD33 gene expression plot
    for name in DCS.getHugoName('CD33'):
        DCS.makeIndividualGeneExpressionPlot(name)

    # Further analysis can be done on cell types of interest, e.g. here 'T cell' and
↪'B cell'.
    # Let's create a new instance of DigitalCellSorter to run "sub-analysis" with it.
    # It is important to disable Quality control, because the low quality cells have
    # already been identified and filtered with DCS.
    # Parameter dataName points to the location processed with DCS.
    DCSsub = DigitalCellSorter.DigitalCellSorter(dataName='BM1',
                                                 nClusters=10,
                                                 doQualityControl=False,
                                                 layout='PHATE',
                                                 subclusteringName='T cell')

    # Modify a few other attributes
    DCSsub.saveDir = os.path.join(here, 'output', 'BM1', 'subclustering T cell', '')
    DCSsub.geneListFileName = os.path.join(here, 'docs', 'examples', 'CIBERSORT_T_SUB.
↪xlsx')

    # Get index of T cells
    indexOfTcells = DCS.getCells(celltype='T cell')

    # Get expression of these T cells using their index
    df_expr = DCS.getExprOfCells(indexOfTcells)

    # Insert expression data into DCSsub
    DCSsub.prepare(df_expr)

    # Process subtype 'T cell'
    DCSsub.process(dataIsNormalized=True)

    # Load marker genes and annotate cells
    DCSsub.annotate()

    # Make plots of annotated data
    DCSsub.visualize()
```

CHAPTER

ELEVEN

INDICES AND TABLES

- genindex
- modindex
- search

PYTHON MODULE INDEX

d

DigitalCellSorter.GenericFunctions, 57
DigitalCellSorter.ReadPrepareDataHCA, 64

INDEX

A

`alignSeries()` (*DigitalCellSorter method*), 43
`annotate()` (*DigitalCellSorter method*), 35
`annotateWith_Hopfield_Scheme()` (*DigitalCellSorter method*), 41
`annotateWith_pDCS_Scheme()` (*DigitalCellSorter method*), 40
`annotateWith_ratio_Scheme()` (*DigitalCellSorter method*), 40

B

`batchEffectCorrection()` (*DigitalCellSorter method*), 45

C

`calculateQCmeasures()` (*DigitalCellSorter method*), 45
`calculateV()` (*DigitalCellSorter class method*), 40
`clean()` (*DigitalCellSorter method*), 34
`cluster()` (*DigitalCellSorter method*), 34
`convert()` (*DigitalCellSorter method*), 34
`convertColormap()` (*DigitalCellSorter class method*), 42
`createReverseDictionary()` (*DigitalCellSorter method*), 43

D

`df_expr()` (*DigitalCellSorter property*), 33
`DigitalCellSorter` (*class in DigitalCellSorter.core*), 29
`DigitalCellSorter.GenericFunctions` (*module*), 57
`DigitalCellSorter.ReadPrepareDataHCA` (*module*), 64

E

`extractFromZipOfGz()` (*in module DigitalCellSorter.GenericFunctions*), 58

F

`fileHDFpath()` (*DigitalCellSorter property*), 33

G

`geneListFileName()` (*DigitalCellSorter property*), 33
`getAnomalyScores()` (*DigitalCellSorter method*), 37
`getCells()` (*DigitalCellSorter method*), 38
`getCountsDataframe()` (*DigitalCellSorter method*), 39
`getDataframeByDonorID()` (*in module DigitalCellSorter.ReadPrepareDataHCA*), 65
`getElapsedTime()` (*in module DigitalCellSorter.GenericFunctions*), 58
`getExprOfCells()` (*DigitalCellSorter method*), 38
`getExprOfGene()` (*DigitalCellSorter method*), 38
`getHCAdataByURL()` (*in module DigitalCellSorter.ReadPrepareDataHCA*), 65
`getHugoName()` (*DigitalCellSorter method*), 37
`getIndexOfGoodQualityCells()` (*DigitalCellSorter method*), 39
`getNewMarkerGenes()` (*DigitalCellSorter method*), 40
`getQualityControlCutoff()` (*DigitalCellSorter method*), 39
`getStartTime()` (*in module DigitalCellSorter.GenericFunctions*), 57
`getSubnetworkOfPCN()` (*DigitalCellSorter method*), 43

I

`internalMakeMarkerSubplots()` (*VisualizationFunctions method*), 48

K

`KeyInFile()` (*DigitalCellSorter class method*), 42

L

`loadAnnotatedLabels()` (*DigitalCellSorter method*), 44
`loadExpressionData()` (*DigitalCellSorter method*), 44

M

makeAnnotationResultsMatrixPlot() (*VisualizationFunctions method*), 53
makeAnomalyScoresPlot() (*DigitalCellSorter method*), 36
makeCellMarkersPiePlot() (*VisualizationFunctions method*), 47
makeHistogramNullDistributionPlot() (*VisualizationFunctions method*), 50
makeHopfieldLandscapePlot() (*DigitalCellSorter method*), 37
makeIndividualGeneExpressionPlot() (*DigitalCellSorter method*), 37
makeIndividualGeneTtestPlot() (*DigitalCellSorter method*), 36
makeMarkerExpressionPlot() (*VisualizationFunctions method*), 55
makeMarkerSubplots() (*DigitalCellSorter method*), 36
makePlotOfNewMarkers() (*VisualizationFunctions method*), 56
makeProjectionPlot() (*VisualizationFunctions method*), 48
makeProjectionPlotAnnotated() (*DigitalCellSorter method*), 35
makeProjectionPlotByBatches() (*DigitalCellSorter method*), 35
makeProjectionPlotByClusters() (*DigitalCellSorter method*), 36
makeProjectionPlotsQualityControl() (*DigitalCellSorter method*), 36
makeQualityControlHistogramPlot() (*VisualizationFunctions method*), 49
makeSankeyDiagram() (*VisualizationFunctions method*), 52
makeStackedBarplot() (*VisualizationFunctions method*), 53
makeTtestPlot() (*VisualizationFunctions method*), 55
mergeIndexDuplicates() (*DigitalCellSorter method*), 44

N

normalize() (*DigitalCellSorter method*), 34

P

prepare() (*DigitalCellSorter method*), 33
PrepareDataOnePatient_PREVIEW_DATASET() (*in module DigitalCellSorter.ReadPrepareDataHCA*), 66
prepareDemo5kData() (*in module DigitalCellSorter.ReadPrepareDataHCA*), 66
prepareMarkers() (*DigitalCellSorter method*), 44
process() (*DigitalCellSorter method*), 35
project() (*DigitalCellSorter method*), 34
propagateHopfield() (*DigitalCellSorter method*), 41

Q

qualityControl() (*DigitalCellSorter method*), 45

R

read() (*in module DigitalCellSorter.GenericFunctions*), 57
readMarkerFile() (*DigitalCellSorter method*), 43
readMTXdata() (*in module DigitalCellSorter.core*), 63
recordAnnotationResults() (*DigitalCellSorter method*), 41
recordExpressionData() (*DigitalCellSorter method*), 44
recordFilesOfIndividualDonors() (*in module DigitalCellSorter.ReadPrepareDataHCA*), 65

S

saveDir() (*DigitalCellSorter property*), 33

T

timeMark() (*in module DigitalCellSorter.GenericFunctions*), 57

V

visualize() (*DigitalCellSorter method*), 35

W

write() (*in module DigitalCellSorter.GenericFunctions*), 57

Z

zScoreOfSeries() (*DigitalCellSorter class method*), 42
